# Inhibitory control hinders habit change

**DOI:** 10.1038/s41598-022-11971-6

**Published:** 2022-05-18

**Authors:** Kata Horváth, Dezso Nemeth, Karolina Janacsek

**Affiliations:** 1 Doctoral School of Psychology, ELTE Eötvös Loránd University, Izabella utca 46, 1064 Budapest, Hungary; 2 Institute of Psychology, ELTE Eötvös Loránd University, Izabella utca 46, 1064 Budapest, Hungary; 3 Brain, Memory and Language Research Group, Institute of Cognitive Neuroscience and Psychology, Research Centre for Natural Sciences, Magyar tudósok körútja 2, 1117 Budapest, Hungary; 4 Department of Cognitive Science, Lund University, Helgonavägen 3, 22100 Lund, Sweden; 5 Lyon Neuroscience Research Center, INSERM, CNRS, Centre Hospitalier Le Vinatier, Université de Lyon, Bâtiment 462, Neurocampus 95 boulevard Pinel, 69675 Bron, Lyon, France; 6 Faculty of Education, Health and Human Sciences, School of Human Sciences, Centre for Thinking and Learning, Institute for Lifecourse Development, University of Greenwich, 150 Dreadnought, Park Row, London, SE10 9LS UK

**Keywords:** Psychology and behaviour, Human behaviour

## Abstract

Our habits constantly influence the environment, often in negative ways that amplify global environmental and health risks. Hence, change is urgent. To facilitate habit change, inhibiting unwanted behaviors appears to be a natural human reaction. Here, we use a novel experimental design to test how inhibitory control affects two key components of changing (rewiring) habit-like behaviors in healthy humans: the acquisition of new habit-like behavior and the simultaneous unlearning of an old one. We found that, while the new behavior was acquired, the old behavior persisted and coexisted with the new. Critically, inhibition hindered both overcoming the old behavior and establishing the new one. Our findings highlight that suppressing unwanted behaviors is not only ineffective but may even further strengthen them. Meanwhile, actively engaging in a preferred behavior appears indispensable for its successful acquisition. Our design could be used to uncover how new approaches affect the cognitive basis of changing habit-like behaviors.

## Introduction

Our automatic, habitual behaviors are constantly challenged. The ongoing threats from environmental and health disasters^1,2^ force us to alter dangerous and unsustainable behaviors, and to replace them with safer, sustainable ones. To achieve this, it is crucial to understand how habits form and change in the healthy human mind^3^.

Habits are traditionally defined as automatic stimulus–response links that are insensitive to the outcome value of the response (as opposed to goal-directed behaviors), by non-human animal studies^4,5^. Previous research aimed at directly translating this definition to measuring habits in humans has repeatedly failed (for recent successful attempts, see^6,7^). Alternatively, human habits can be defined as more complex behaviors that are characterized by a collection of behavioral attributes: they are acquired via associative learning processes gradually over an extended period of practice, often without conscious awareness, and once developed, they can be performed with little thought or attention (i.e., automatically; for more details see the “Behavioral and neural characteristics of habit learning across human and animal studies” section in the Supplementary Information)^8–13^. During habit change, new associations are learned to replace old ones, suggesting that overcoming old habits and developing new habits share the same learning process^14,15^. Aspects of habit change have been widely studied in clinical and health settings (e.g., addiction), in non-human animals, and in relation to reward-related behavior (e.g., extinction and counterconditioning)^16,17^. This research has extensively characterized the computational and neural underpinnings of how simple stimulus–response(–reward) associations contribute to habit formation and change. However, it remains poorly understood how habit change occurs in healthy humans when more complex associations (i.e., when not only the current stimulus influences the response but a sequence of preceding stimuli) are learned and modified without explicit rewards^18^. These features more closely resemble habit change in daily life; therefore, identifying the cognitive changes that occur during habit change in these contexts could significantly broaden our understanding in this field.

A recent study using self-reported measures in healthy individuals found that increasing the frequency of new, sustainable behaviors (i.e., forming sustainable habits) was perceived to be more feasible than reducing old, unsustainable ones^19^. When participants imagined reducing unsustainable behaviors, the right dorsolateral prefrontal cortex—a key brain region for inhibitory-control processes—was activated. This finding suggests that inhibiting old, unsustainable behaviors may be a natural reaction when attempting to change habits. Research on habit change in everyday settings has also implicated the role of effortful inhibition and self-control in overcoming unwanted behaviors^18,20^. Importantly, however, how inhibitory control—the ability to suppress prepotent but unwanted actions, thoughts, or emotions^21,22^—affects habit change when complex associations need to be modified has not yet been directly probed in a controlled experimental setting in healthy humans.

**Figure 1:**
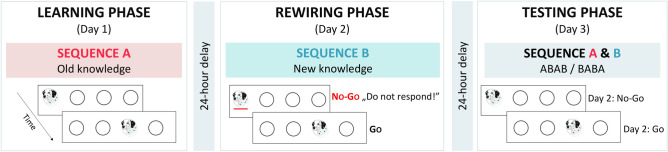
Design of the experiment. The experiment consisted of three phases, each separated by 24-h delays. During the Learning phase, participants extensively practiced a four-choice visuomotor reaction time task over 3600 trials, divided into three periods. In this task, a stimulus appeared in one of four horizontally arranged circles on the screen, and participants were asked to respond as quickly and accurately as they could using a response box. The associations of Sequence A (referred to as old knowledge) were acquired in this phase. Then during the Rewiring phase, a structural change was introduced to the task with Sequence B to prompt the rewiring of the old knowledge by acquiring the associations of this new sequence (referred to as new knowledge). Additionally, to engage participants’ inhibitory control processes in this phase, they were asked to suppress their responses on some trials (stimuli underlined with a red line during the task, No-go trials), but could respond on other (Go) trials. This phase also consisted of 3600 trials, divided into three periods. In the Testing phase, using a shorter version of the task, knowledge of both sequences was probed in a counterbalanced order (ABAB or BABA on the figure, where A and B refer to the sequence used in the Learning and Rewiring phases, respectively). Here, responses were allowed on all trials, including previously suppressed No-go trials, to assess the effect of inhibitory control on rewiring. The stimulus was taken from the public domain (retrieved on 26/09/2017 from: www.pixabay.com).

Here we created a novel experimental design to test how inhibitory control affects two key components of changing habit-like behaviors: the acquisition of new complex associations and the simultaneous unlearning of old ones, in a neutral environment (i.e., without explicit rewards). Learning processes were examined via *rewiring*, whereby structural changes in the experimental task promoted the acquisition of new associations in place of old ones^23^. To test the rewiring of the initially acquired knowledge (henceforth referred to as old knowledge), we first needed to ensure that this knowledge was indeed acquired. This was assessed during the Learning phase, where 33 healthy young adults underwent an extensive practice on a visuomotor, four-choice reaction time task^24–26^ (Fig. 1). Unbeknownst to them, location of the visual stimuli followed a predetermined sequential order that alternated with randomly chosen locations, resulting in some runs of three consecutive trials (referred to as triplets) being more probable than others. This enabled us to track the initial acquisition of complex associations continuously.

**Figure 2:**
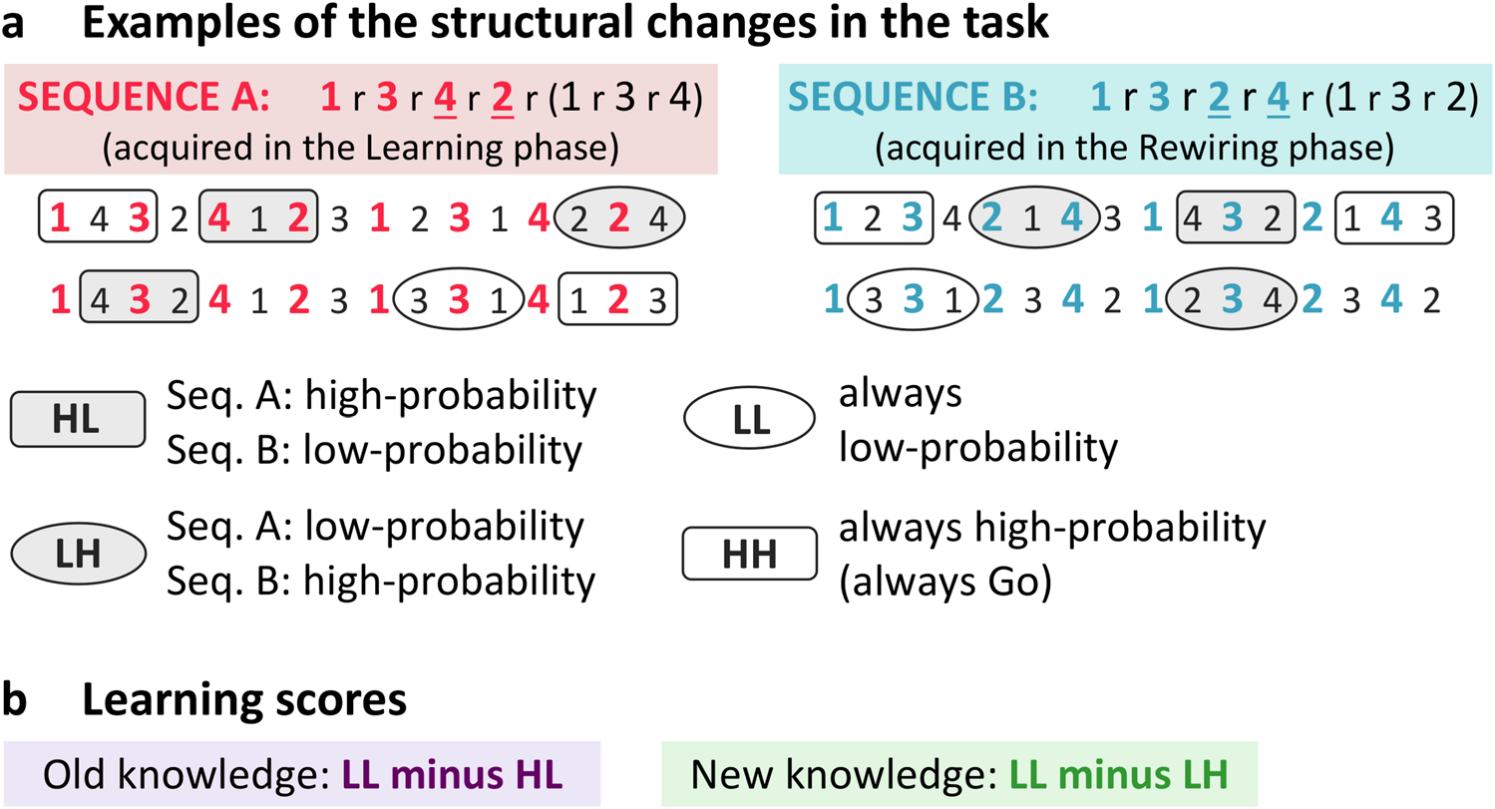
Task structure and measures of learning in the experiment. (**a**) Locations of the visual stimuli followed a predetermined sequential order (1 through 4 on the figure indicate the four horizontally arranged locations on the screen) that alternated with randomly chosen locations (indicated by r) out of the four possible ones. Example sequences are shown on the figure; overall, pairs of six unique sequences were used in a counterbalanced order. Due to the alternating sequence structure, some runs of three consecutive trials were more probable than others (referred to as high- vs. low-probability triplets, respectively)^29^. An example of the difference between Sequence A and Sequence B used in the Learning and Rewiring phases, respectively, is shown by the underlined numbers. Due to this structural change in the task, the probability of some triplets changed from the Learning phase to the Rewiring phase: 75% of the initially high-probability triplets became low-probability (HL trials; thus, the first letter refers to the triplet probability in Sequence A, while the second letter refers to the probability of the same triplet in Sequence B) and were replaced by new high-probability triplets that were initially low-probability (LH trials). Additionally, the occurrence probability of some triplets remained constant: either being low-probability (LL trials) or high-probability (HH trials) in both phases (for further details see “Methods” section). (**b**) Learning scores were calculated as differences in response times to trials with changed (LH or HL) versus unchanged occurrence probabilities (LL or HH). This enabled us to assess how participants initially acquired the associations of Sequence A, and then updated their knowledge when practicing Sequence B. For example, we expected similarly slow responses to LH and LL trials in the Learning phase (as both were low-probability) but then faster responses to LH than LL in the Rewiring phase, indicating the acquisition of the more probable associations of Sequence B in this phase. Please note that all HH trials were Go during the Rewiring phase (for further details, see the “Supplementary methods” section in Supplementary Information). Consequently, learning scores involving LL trials were the primary measures of interest as these could be used to assess the effect of inhibitory control on rewiring (by contrasting learning scores calculated on those trials that were Go vs. No-go in the Testing phase).

This old knowledge was then challenged in the Rewiring phase, in which a structural change was introduced to the task. Seventy-five percent of originally high-probability triplets became low-probability (denoted as HL trials) and were replaced by new high-probability triplets (that were originally low-probability, denoted as LH trials; see Fig. 2a and, for further details, the “Supplementary methods” section in the Supplementary Information), prompting the rewiring of the old knowledge. Thus, participants needed to unlearn most of what they acquired in the Learning phase as it was no longer relevant, and simultaneously acquire new associations from the partially changed sequence (henceforth referred to as new knowledge). Additionally, participants were asked to actively inhibit responses on some trials to engage their inhibitory control processes in this phase^27,28^. Then, both the old and new knowledge was assessed in the Testing phase. Here, responses were allowed on all trials, including those in which participants inhibited their responses during rewiring, to probe how inhibition affected their (un)learning processes. Using this carefully controlled experimental setting, we were able to directly examine how inhibitory control affects the (un)learning of complex associations that underlie automatic habit-like behaviors in healthy adults.

### Initial acquisition and subsequent unlearning of associations that were no longer relevant due to the structural change in the task

**Figure 3:**
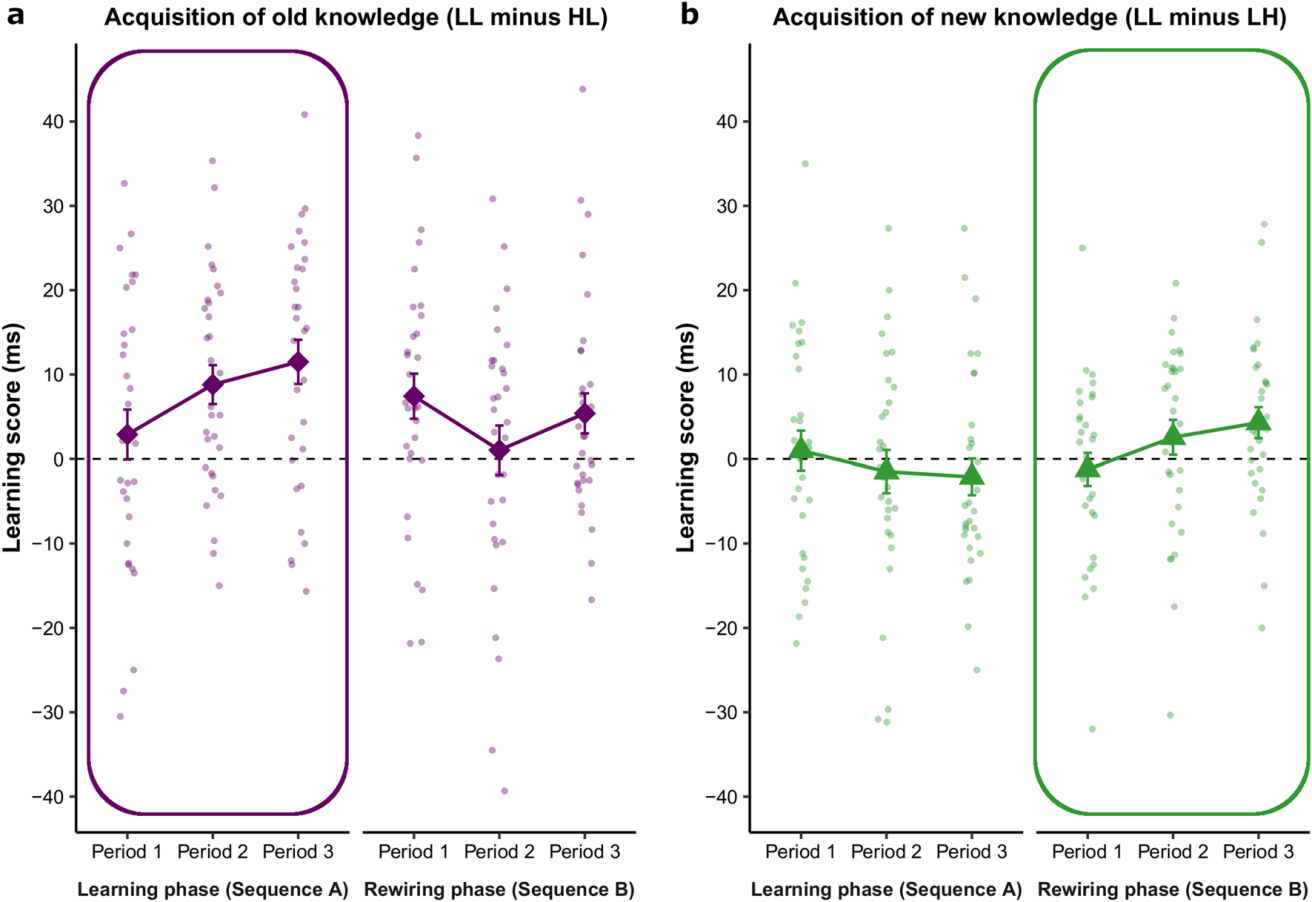
Learning trajectories of old and new knowledge in the learning and rewiring phases. The circled panels indicate the experimental phase in which higher learning scores were expected based on the probability of the trial types of comparison. For example, for the old knowledge (‘LL minus HL’ learning score), higher learning scores were expected in the Learning phase as trials with high vs. low triplet occurrence probability were contrasted here (‘LL minus HL’, underlined letters indicating probabilities of the current comparison; see also Fig. 2). (**a**) Participants successfully acquired the old knowledge (associations of Sequence A) in the Learning phase, indicated by gradually increasing learning scores. Then they at least partially unlearned this old knowledge in the Rewiring phase. (**b**) The new knowledge (associations of Sequence B) was gradually acquired in the Rewiring phase. (Since these associations were all low-probability in Sequence A, no learning was expected for them in the Learning phase). Please note that learning scores used in these analyses were calculated for Go trials only because no reaction times were collected for No-go trials in the Rewiring phase. Error bars represent Standard Error of the Mean (SEM).

Learning successfully occurred in the Learning phase (Fig. 3a, circled area): participants showed increasingly higher learning scores (‘LL minus HL’, underlined letters indicating the triplet probabilities of the current comparison), reflecting faster responses to trials that were high-probability in Sequence A compared to low-probability ones (for raw RTs see Fig. S1a). This old knowledge was then partially unlearned during the Rewiring phase (Fig. 3a, non-circled area), in which originally high-probability trials became less probable (‘LL minus HL’; thus, both trial types compared were low-probability in Sequence B). The different time course of (un)learning across the two phases is indicated by the significant Phase × Period interaction (*F*(2, 60) = 5.70, *p* = 0.005, η_*p*_^2^ = 0.160). Specifically, participants gradually acquired the associations of Sequence A (Period 1 vs. Period 3: *p* = 0.002, Cohen’s *d* = 0.60, BF_01_ = 0.059), with learning scores differing significantly from zero in Period 2 (*p* = 0.001, Cohen’s *d* = 0.70, BF_01_ = 0.020) and Period 3 (*p* < 0.001, Cohen’s *d* = 0.79, BF_01_ = 0.005) of the Learning phase. In the Rewiring phase, learning scores started to slightly decrease (Period 1 vs. Period 2: *p* = 0.050, Cohen’s *d* = 0.37, BF_01_ = 0.840; all other *p*s ≥ 0.178, Cohen’s *d*s ≤ 0.25, BF_01_s ≥ 2.210), reaching zero in Period 2 (*p* = 0.726, Cohen’s *d* = 0.06, BF_01_ = 4.926), and then slightly bounced back in Period 3 (*p* = 0.030, Cohen’s *d* = 0.41, BF_01_ = 0.564). The main effects were not significant (Phase: *F*(1, 30) = 1.46, *p* = 0.237, η_*p*_^2^ = 0.046; Period: *F*(2, 60) = 1.74, *p* = 0.184, η_*p*_^2^ = 0.055). Overall, participants successfully acquired the associations of Sequence A in the Learning phase and could at least partially unlearn this knowledge in the Rewiring phase.

### Acquisition of new knowledge after structural change in the task

Learning of the new sequence occurred in the Rewiring phase (Fig. 3b, circled area): participants showed increasingly higher learning scores (‘LL minus LH’), indicating faster responses to trials that were high-probability in Sequence B compared to low-probability ones (for raw RTs see Fig. S1a). Note that these associations were all low-probability in Sequence A; therefore, no learning was expected for them in the Learning phase (‘LL minus LH’). The different time course of learning across the two phases was revealed by the significant Phase × Period interaction (*F*(2, 60) = 3.89, *p* = 0.026, η_*p*_^2^ = 0.115). Specifically, performance did not change significantly during the Learning phase (pair-wise comparisons of periods: all *p*s ≥ 0.282, Cohen’s *d*s ≤ 0.20, BF_01_s ≥ 3.019) and did not differ significantly from zero (all *p*s > 0.339, Cohen’s *d*s ≤ 0.17, BF_01_s ≥ 3.390). In the Rewiring phase, learning scores increased from Period 1 to Period 3 (*p* = 0.019, Cohen’s *d* = 0.44, BF_01_ = 0.390) and became greater than zero by the end of the task (Period 3: *p* = 0.026, Cohen’s *d* = 0.42, BF_01_ = 0.503). The main effects were not significant (Phase: *F*(1, 30) = 1.60, *p* = 0.216, η_*p*_^2^ = 0.051; Period: *F*(2, 60) = 0.20, *p* = 0.820, η_*p*_^2^ = 0.007). In summary, these results confirm that participants acquired the associations of the new sequence after the structural change in the task. For a further analysis on how the acquisition of new knowledge compares with the initial learning process, see the Supplementary Information.

### How did the inhibition of responses during rewiring affect the old knowledge?

In the Testing phase, we probed whether the old knowledge (using the ‘LL minus HL’ learning score) was expressed both in the old testing context (when the order of stimulus presentation followed Sequence A) and the new one (when stimulus presentation followed Sequence B; see also Fig. 1 for design). Knowledge on the previously Go and No-go trials was contrasted in both testing contexts. As expected, learning scores were significantly higher when tested on Sequence A than on Sequence B (main effect of Sequence: *F*(1, 30) = 10.11, *p* = 0.003, η_*p*_^2^ = 0.252), regardless of the Go/No-go manipulation. At the same time, they were significantly above zero in both contexts, indicating that the old knowledge was expressed not only in its original context (Sequence A; ‘LL minus HL’, *p* < 0.001, Cohen’s *d* = 1.22, BF_01_ = 1.845^e−5^) but also in the new one (Sequence B; ‘LL minus HL’, *p* < 0.001, Cohen’s *d* = 0.71, BF_01_ = 0.147), where it was no longer relevant.

**Figure 4:**
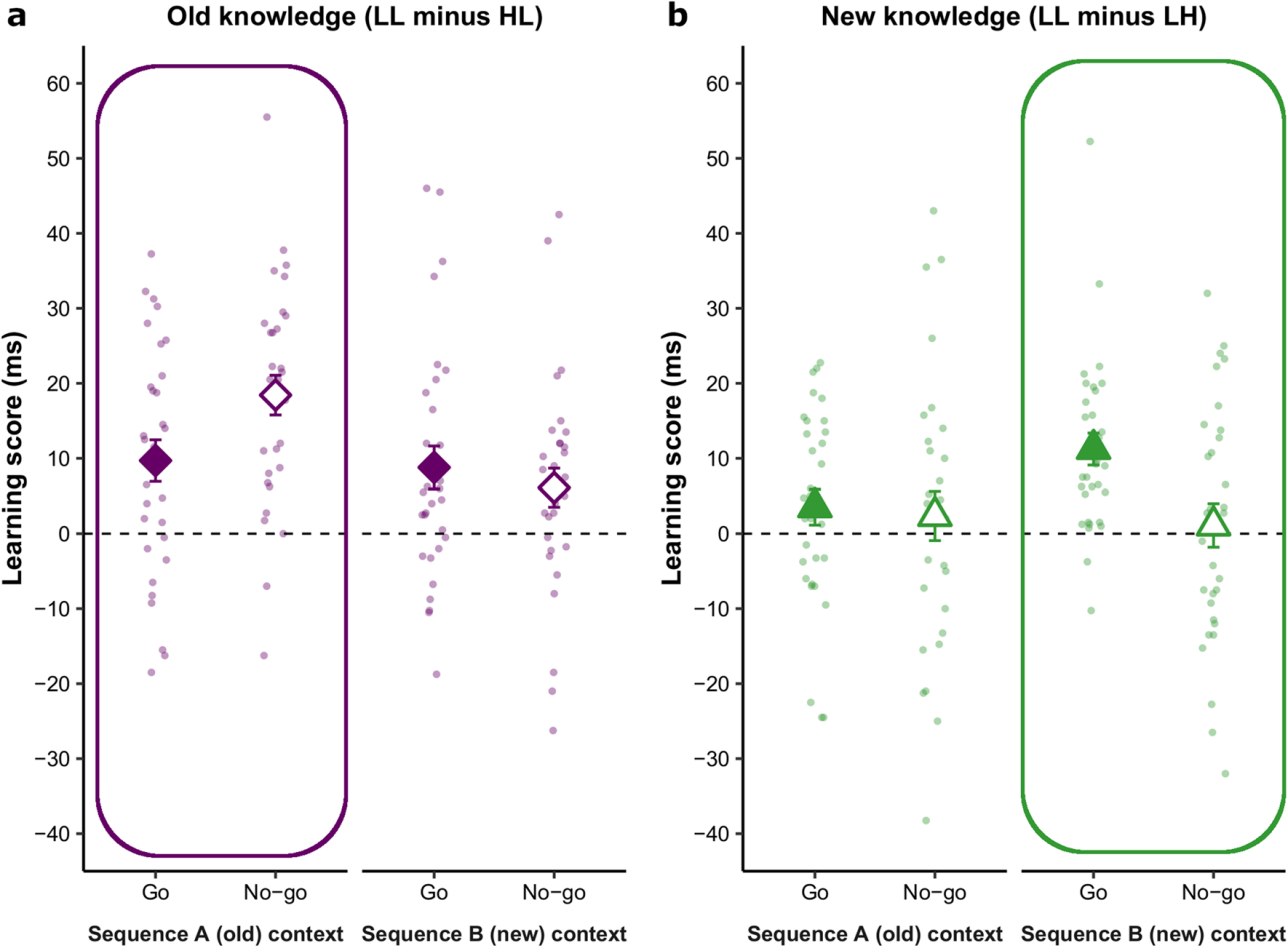
The effect of inhibitory control on old and new knowledge as revealed by performance in the Testing phase. The circled panels indicate the testing context (task version with Sequence A or B) in which higher learning scores were expected. For example, for the new knowledge (‘LL minus LH’ learning score), higher learning scores were expected in the new context (when stimulus presentation order followed Sequence B), since trials with high vs. low triplet occurrence probabilities were contrasted here (‘LL minus LH’, underlined letters indicating probabilities of the current comparison; see also Fig. 2). (**a**) Old knowledge. When tested on Sequence A (the original, old context), participants showed significant above-zero performance on Go and No-go trials, with significantly higher learning scores for the latter. This suggests that the old knowledge was present, and inhibiting responses during rewiring strengthened, instead of facilitated, its unlearning. When tested on Sequence B (the new context), participants exhibited similar, significantly above-zero learning scores on Go and No-go trials, suggesting that old knowledge was expressed even when it was not relevant, irrespective of whether responses were inhibited during rewiring. (**b**) New knowledge. When tested on Sequence B (the relevant, new context), participants showed significant above-zero learning scores only on Go trials and these learning scores differed significantly from those on No-go trials, indicating that new knowledge could be expressed only if responses were allowed to the relevant stimuli during rewiring. Thus, actively engaging in the new behavior-to-be-learned seemed essential for acquiring (and subsequently accessing) the new knowledge. When tested on Sequence A, participants’ learning scores did not differ significantly from zero either on Go or No-go trials. This was expected since contrasted trials were all low-probability in Sequence A (‘LL minus LH’). Error bars represent SEM.

Crucially, the magnitude of learning scores depended both on the testing context (Sequence A vs. B) and whether responses were inhibited during rewiring (Go vs. No-go trials), as indicated by the significant Sequence × Inhibition interaction (*F*(1, 30) = 11.81, *p* = 0.002, η_*p*_^2^ = 0.282). When tested on Sequence A (Fig. 4a, circled area), learning scores were significantly above zero on Go and No-go trials (*p* = 0.001, Cohen’s *d* = 0.63, BF_01_ = 0.042;* p* < 0.001, Cohen’s *d* = 1.25, BF_01_ = 6.508^e−6^, respectively) and somewhat greater for the latter (*p* = 0.018, Cohen’s *d* = 0.45, BF_01_ = 0.360). This suggests that, instead of facilitating the unlearning process, inhibition potentially strengthened the expression of old knowledge in the old context. When tested on Sequence B (Fig. 4a, non-circled area), learning scores did not differ significantly on Go and No-go trials (*p* = 0.500, Cohen’s *d* = 0.12, BF_01_ = 4.210). Importantly, participants performed significantly above zero on both (Go trials: *p* = 0.004, Cohen’s *d* = 0.55, BF_01_ = 0.112; No-go trials: *p* = 0.025, Cohen’s *d* = 0.42, BF_01_ = 0.486), again indicating that old knowledge was expressed even when it was not relevant, irrespective of whether responses were inhibited during rewiring.

From another perspective, learning scores on Go trials did not differ significantly across testing contexts (*p* = 0.735, Cohen’s *d* = 0.06, BF_01_ = 4.945). In contrast, learning scores on No-go trials were significantly higher when tested on Sequence A than Sequence B (*p* < 0.001, Cohen’s *d* = 0.83, BF_01_ = 0.003), suggesting that the detrimental effect of inhibition (boosting, instead of decreasing old knowledge) was greater in the old context than the new one. The main effect of Inhibition was not significant (*F*(1, 30) = 0.83, *p* = 0.37, η_*p*_^2^ = 0.027). Overall, these results highlight the persistence of old knowledge across testing contexts and suggest a detrimental effect of the inhibition of responses during rewiring.

### How did the inhibition of responses during rewiring affect the new knowledge?

In the Testing phase, new knowledge (‘LL minus LH’) was differentially expressed depending both on the testing context (Sequence A vs. B) and whether responses were inhibited during the Rewiring phase (Go vs. No-go trials), indicated by the significant Sequence × Inhibition interaction (*F*(1, 30) = 4.20, *p* = 0.049, η_*p*_^2^ = 0.123). When tested on Sequence A, learning scores did not differ significantly from zero either on Go or No-go trials (*p* = 0.150, Cohen’s *d* = 0.27, BF_01_ = 1.956 and *p* = 0.478, Cohen’s *d* = 0.13, BF_01_ = 4.115 respectively; Go vs. No-go: *p* = 0.780, Cohen’s *d* = 0.05, BF_01_ = 5.030). This was expected because the contrasted trials were all low-probability in Sequence A (‘LL minus LH’; Fig. 4b, non-circled area). When tested on Sequence B (the context relevant to new knowledge; ‘LL minus LH’, Fig. 4b, circled area), learning scores were significantly above zero on Go trials (*p* < 0.001, Cohen’s *d* = 0.95, BF_01_ = 5.084^e−4^) but not on No-go trials (*p* = 0.710, Cohen’s *d* = 0.07, BF_01_ = 4.889). The difference between learning scores on Go vs. No-Go trials was significant (*p* = 0.003, Cohen’s *d* = 0.58, BF_01_ = 0.078). This indicates that participants could successfully express new knowledge only if permitted to respond to the relevant stimuli during rewiring.

Conversely, although performance on No-go trials did not differ significantly across testing contexts (*p* = 0.694, Cohen’s *d* = 0.07, BF_01_ = 4.853), performance on Go trials did: learning scores were significantly higher when tested on Sequence B vs. on Sequence A (*p* = 0.009, Cohen’s *d* = 0.50, BF_01_ = 0.201). This suggests that newly acquired knowledge was successfully expressed only in its relevant context. The main effects were not significant (Sequence: *F*(1, 30) = 2.56, *p* = 0.120, η_*p*_^2^ = 0.078; Inhibition: *F*(1, 30) = 3.74, *p* = 0.063, η_*p*_^2^ = 0.111).

Overall, these findings indicate that participants successfully acquired the new knowledge on Go trials (for which responses were allowed during rewiring) and could express it in the appropriate context (i.e., when tested on Sequence B). At the same time, poorer performance on No-go trials suggests that *actively* engaging in the new behavior-to-be-learned may be essential for acquiring new associations and, consequently, for habit change.

## Discussion

Changing habits is challenging^3^, but as threats of environmental and health disasters rapidly increase across the world^1,2^, it is more important than ever to find effective ways to succeed. To do so, it is vital that we gain a thorough understanding of how habits form and change. Previous research has extensively focused on non-human animals, reward-related behaviors, and clinical populations in humans, and characterized how simple stimulus–response(-reward) associations contribute to habit formation and change^16,17^. However, it is poorly understood how habit change occurs in healthy humans when more complex associations (i.e., when not only the current stimulus influences the response but a sequence of preceding stimuli) are learned and modified without explicit rewards. These features more closely resemble how habits form and change in daily life. Therefore, by probing how healthy human adults can form and rewire complex associations without explicit rewards, the present study can significantly contribute to our understanding of the key cognitive processes involved in habit change.

Using these features, we created a novel experimental design to test a widely held belief that inhibitory control could promote habit change^19,20^. In this design, we could test the acquisition of new habit-like behaviors and the simultaneous unlearning of old ones, and how inhibitory control affected both. Crucially, following the rewiring process, we probed both the old and new knowledge across original (old) and new testing contexts, and on those trials in which responses were or were not allowed previously, to reveal how inhibitory control affected the entire process of rewiring. We found that inhibiting responses had a detrimental effect on overcoming the old knowledge and establishing the new: old knowledge was retained and expressed not only in its original context but also in the new one; moreover, components of knowledge that were previously inhibited appeared to be even strengthened in the old context (Fig. 4a). New knowledge was expressed only in the new context and for those components to which responses were allowed (Fig. 4b), suggesting that actively engaging in the behavior-to-be-learned may be indispensable for successfully changing habit-like behaviors.

Our findings revealed the persistence of old knowledge in both the old and new contexts, irrespective of whether components were inhibited during rewiring. Recently, a new line of research on the competition between habitual and goal-directed responses following changes in stimulus–outcome^6^ or stimulus–response^7^ associations has revealed a similar persistence effect. Specifically, following extended training and under time pressure—shown to favor the expression of habit-like behaviors—reaction times increased for the goal-directed (desired) responses and participants committed a large proportion of habitual (undesired) errors. These findings highlight that habitual (“old”) and goal-directed (“new”) associations are in conflict during response selection, and, together with the present study, suggest that undesirable habit-like behaviors may exert their influence even if the desired behavior is ultimately executed (see previously not inhibited components of new knowledge exhibited successfully in their corresponding [new] context).

Inhibiting responses during rewiring shows some similarities with extinction learning, whereby the well-established, habit-like behavior (response) fades over time as the previously conditioned stimulus is repeatedly presented without any reinforcer^14,27,30,31^. Following extinction, relapse—reoccurrence of the extinct behavior/response—is often observed^17,32^. Our findings in the Testing phase show that relapse can occur not only when human participants encounter the original context e.g.^33,34^ (akin to extinction learning studies) but also in the new context. This suggests that inhibiting unwanted behavior in everyday situations is ineffective in changing habits e.g.^35^. Importantly, as opposed to the typical settings in extinction studies, our results were observed without any explicit rewards being involved in either learning or rewiring, and alternative associations could be learned to replace the old ones (instead of just unlearning them). The persistence of old knowledge despite these characteristics suggests that extinction studies may underestimate the effect of suppressing old behaviors in habit change.

Our findings also suggest that inhibiting responses may even further strengthen cognitive representations underlying the original behavior we want to replace, resulting in a rebound effect. This is based on participants exhibiting higher learning scores on the previously inhibited components of old knowledge (‘LL minus HL’, No-go) compared to those that were not inhibited (‘LL minus HL’, Go), when tested in the old context (Sequence A). Note, however, that the effect size for this finding was slightly smaller (Cohen’s *d* = 0.45) than the one used in the a priori calculations (a Cohen’s *d* of 0.50; see the “Estimation of required sample size” section in the Supplementary Information) and, consequently, the post-hoc power appeared somewhat lower than expected (power = 0.68 for two-tailed comparisons, instead of the expected 0.80). Therefore, future studies are needed to replicate this rebound effect^6,7^. Beyond the persistence of old knowledge, our design could also reveal that old and new knowledge coexisted in the new context (at least for those trials in which responses were allowed during rewiring). We observed this effect both in reaction time (RT) (reported in the main text) and response accuracy measures (see Supplementary Information). This finding could explain the competition that could occur between old and new behaviors during habit change, and thus serve as the cognitive basis for such competition^27^. To translate these findings to a real-life example, let us suppose that Mary has just moved to Country B. Here, recycling is much more prevalent than her previous residence in Country A, and she has therefore had to start dividing household waste into different bins depending on its material. In this case, the old behavior (throwing all household waste into the same bin) is expected to be gradually unlearned and replaced by the new behavior (dividing waste into separate bins). Despite the decision to change her behavior, it is possible that (i) when Mary re-visits Country A (old context) she reverts to not recycling (relapse of the old behavior), and (ii) even in Country B (new context), she might divide waste on some occasions but not on others (coexistence of old and new behaviors). Furthermore, Mary may, consciously or unconsciously, suppress some aspects of her habitual behavior of not dividing waste, which could exacerbate the above-described behavioral pattern. Since old and new behaviors coexist, and a continuous inhibition of the old behavior may be unsustainable over longer periods, our findings highlight that interventions using other approaches for habit change must be tested (for further discussion see^18,36,37^).

One might argue that our results are driven by an incomplete acquisition of the new knowledge as suggested by data from the Rewiring phase (see also the “How does acquisition of new knowledge compare with the initial learning process?” section in the Supplementary Information). However, some aspects of performance in the Testing phase suggest otherwise. Specifically, direct comparisons of old and new knowledge indicate that, of those trials on which responses were allowed during rewiring, participants could express old and new knowledge at a similar level, both when compared in their respective contexts (i.e., in Sequence A vs. Sequence B, respectively), as well as in the new (Sequence B) context (see “Is the level of the new knowledge comparable to that of the old knowledge in the Testing phase?” section in the Supplementary information). Since a 24-h delay period was included between the Rewiring and the Testing phases in our design, it is likely that consolidation (i.e., stabilization) of memory traces occurred in this period^23^, facilitating the expression of newly acquired knowledge in the Testing phase. Future research should test how rewiring schedules with different durations of practice and different lengths of consolidation periods in-between^38,39^ affect old and new knowledge across testing contexts.

In our experimental design, the duration of training for rewiring and the acquisition of old knowledge was the same. Recent studies showed that while we can acquire associative knowledge relatively quickly, updating it requires more extended practice^40,41^. Likewise, non-human animal studies of behavior change usually apply a non-fixed time window of training, lasting until the animal no longer exhibits signs of the original behavior^42,43^. Note, however, that this would be unfeasible in daily life as we may want to change behaviors that were developed and practiced over years or even decades. Consequently, in real-life examples of habit change, holding all other factors constant, we may expect an even weaker acquisition of new behavior and a stronger persistence of old behavior compared to what we observed in the current study. As the same amount of practice for new, preferred behaviors is unfeasible, new approaches need to be found and tested. Importantly, any such approach will need to track both the unlearning of old behavior and the acquisition of new behavior, as well as subsequently probe their coexistence—akin to the design of the current study.

What other factors should future research of habit change consider? While here, both the old and new knowledge were acquired incidentally (see also results of the free generation and triplet sorting tasks in the Supplementary Information), encouraging intentional processes during rewiring (e.g., providing explicit instructions on what aspects of behavior to change) may be beneficial, albeit potentially temporary^23^. This is consistent with the observation that aspects of learning may be initially accessible to consciousness, however, after extended practice, at least some components of the automatic, habitual behaviors are no longer consciously accessible^8,44^.

The age when habits are acquired and then changed should also be considered. Although how people of different ages perform in habit change are poorly understood, research has shown that children, especially under the age of 12, are better at acquiring new complex associations underlying automatic behaviors, while older adults show significant difficulties in doing so, compared to young adults^45,46^. Our current study focused on young adults; investigating the same aspects of habit change in other age groups would be particularly important given the aging population across the world^47^. Since habit change involves not only unlearning old, unwanted behavior but also acquiring new, preferred behavior, we expect poorer performance and even stronger persistence of old behavior in older adults. Meanwhile, the childhood advantage in acquiring automatic behavior could be extensively utilized: ensuring that sustainable habits are learned in childhood could be key to succeeding in the global race for sustainability. Besides age, other characteristics of the sample should also be considered in the future: notably, the present study investigated educated young adults from the western world (often referred to as WEIRD people^48^), potentially limiting the generalizability of the present findings to a subgroup of the global population.

The present study applied an experimental design that was novel in several respects. First, we could track two key components of changing habit-like behaviors, that is, the acquisition of new knowledge and the simultaneous unlearning of old knowledge within the same task. Second, we investigated complex associations that could be acquired by responding to probability-based relationships between events of a stimulus stream, as opposed to more commonly used simple(r) stimulus–response associations in lab-based tasks. Third, we tested rewiring and the role of inhibitory control without explicit rewards or reinforcers, contrary to most human and non-human lab-based studies^27,43^. We considered this important as using rewards could evoke processes that are specifically related to the reward itself and would change the motivational/emotional aspects of habit change, possibly confounding the measurement of reward-independent learning processes underlying habit formation and change. These characteristics allowed us to more closely model how humans naturally develop habit-like behaviors^44,49,50^ and test how inhibitory control affects key components of changing such behaviors. Nevertheless, as there are numerous experimental tasks to test habit learning and change, all grasping (at least somewhat) different aspects of these processes (for more details see the “Behavioral and neural characteristics of habit learning across human and animal studies” section in the Supplementary information), further studies are needed to adapt our design to and test the role of inhibitory control in habit change with other tasks as well.

In conclusion, using a novel experimental design, we found that even though it is possible to acquire new habit-like behaviors, a parallel inhibition of the unwanted behavior may be maladaptive and may even strengthen the behavior we want to overcome. Thus, although inhibiting unwanted automatic behavior might be a natural reaction when attempting to replace unwanted, unsustainable habits with preferred, sustainable ones^19^, employing inhibitory control during habit change seems to have no beneficial effect on this process. The design developed here could be used to test new approaches to habit change, thereby uncovering how they affect the cognitive basis of old and new habit-like behaviors, independent of reward effects, in healthy adults and other populations. This can help us develop new intervention techniques for habit change and thereby create more adequate policies, improving our odds of replacing unwanted automatic behaviors with preferred ones.

## Methods

### Participants

Thirty-three healthy undergraduate students participated in the experiment. They were attendees of a non-compulsory university course where course credits could be obtained by participating in scientific experiments and were randomly assigned to the present study. The sample size was determined based on previous studies using similar experimental tasks in within-subject designs^23,24^ (for details, see the “Estimation of required sample size” section in Supplementary Information). Participants had normal or corrected-to-normal vision. None of them reported a history of any psychiatric or neurological condition, or substance use. One participant dropped out of the experiment due to technical errors during data collection. Another participant was excluded due to consistent outlier performance (± 2 SDs) on RT measures throughout the experiment. Therefore, 31 participants remained in the final sample (*M*_Age_ = 21.1 years, *SD*_Age_ = 2.15 years, *M*_Education_ = 14.2 years, *SD*_Education_ = 1.69 years, 29 females). They performed in the normal range on standard neuropsychological tests [Digit Span task^51,52^: *M* = 7.8, *SD* = 1.29; Counting Span task^53,54^: *M* = 3.7, *SD* = 0.70]. Prior to their inclusion in the study, participants provided informed consent to the procedure as approved by the Research Ethics Committee of the Eötvös Loránd University, Budapest, Hungary (Ref. no.: 2018/192). The study was conducted in accordance with the Declaration of Helsinki, and participants received course credits for taking part in the experiment.

### Design

The experiment consisted of three phases, each separated by a 24-h (± 1 h) offline delay (Fig. 1). During the Learning phase (Day 1), participants performed a widely used and reliable^55^ four-choice visuomotor reaction time task called Alternating Serial Reaction Time (ASRT) task^29,56^, in which they acquired the associations of Sequence A. This is referred to as old knowledge throughout the paper. During the Rewiring phase (Day 2), a structural change was implemented in the task by introducing Sequence B. This change prompted the rewiring of old knowledge by acquiring associations of the new sequence. This is referred to as new knowledge*.* In this phase, participants were asked to suppress their responses on some trials (stimuli underlined with a red line during the task; No-go trials), while they were allowed to respond on other trials (Go trials). During the Testing phase (Day 3), participants completed a shorter version of the task, and performance was tested on both Sequence A and Sequence B in a counterbalanced order. In this phase, participants responded on all trials, including the ones that were No-go trials during the Rewiring phase. This enabled us to test how inhibitory control during rewiring affected the unlearning of old associations and the simultaneous acquisition of new associations. Throughout the experiment, participants were informed that they would participate in an experiment assessing reaction times and response accuracy changes over extended practice; thus, both learning and rewiring occurred incidentally^57^. This was chosen because in everyday situations many habits are developed incidentally^18,44^; note the current study aimed to test the role of inhibitory control on (un)learning processes and not the effect of incidental vs. intentional processes on rewiring, for that see^23^. For the detailed description of the ASRT task and the structural changes introduced in the Rewiring phase, see the “Supplementary methods” section in the Supplementary Information.

At the end of the Testing phase, a free generation task and a triplet sorting task were administered to probe whether participants acquired consciously accessible knowledge about the sequence and/or the probability structure of the task using recall- and recognition-based approaches, respectively. Since these tasks were not designed to contrast knowledge gained/rewired on Go vs. No-go trials, they served the sole purpose of testing whether any knowledge throughout the task became consciously accessible; the results are reported in the Supplementary Information for comparability across studies and to support future meta-analytic efforts.

### Statistical analysis

#### Learning phase and rewiring phase

To track the trajectory of the acquisition and unlearning of old knowledge and the simultaneous acquisition of new knowledge, we analyzed the Go trials of these two phases. First, trials were categorized based on whether they were high- or low-probability in the Learning phase (according to Sequence A) and whether they were high- or low-probability subsequently in the Rewiring phase (according to Sequence B). This resulted in four trial types: HL, LH, HH and LL, in which the first letter denotes the probability in the Learning phase and the second letter denotes the probability of the same trial in the Rewiring phase (Fig. 2a; H—high-probability, L—low-probability). Second, data were grouped into three periods, each containing 15–15 ASRT blocks for both phases. Third, for each participant, period, and trial type, median RTs for correctly responded trials were computed.

Fourth, learning scores were computed as differences in response times on trials with changed (LH or HL) versus unchanged occurrence probabilities (LL or HH). Specifically, we expected that participants would become increasingly faster on HL trials during the Learning phase, as compared to the LL trials (for raw RT performance see Fig. S1 in the Supplementary Information), resulting in increasingly higher learning scores (‘LL minus HL’, Fig. 3a) in this phase. This would indicate the acquisition of old knowledge^24,29^. Then, in the Rewiring phase, unlearning of this knowledge would be reflected in smaller/decreasing learning scores as in this case the initially high-probability trials became low-probability. Furthermore, we expected similarly slow responses to LH and LL trials in the Learning phase (reflected in near-zero learning scores) as here both were low-probability, and then faster responses to LH than LL in the Rewiring phase (reflected in increasingly higher/positive learning scores, ‘LL minus LH’, Fig. 3b), indicating the acquisition of new knowledge in this phase. The LL trials served as a baseline for these learning scores as they helped control for general practice effects, while no speed-up was expected on them due to probability-based learning as they were low-probability in both phases.

Finally, repeated-measures analyses of variance (ANOVAs) with Phase (Learning vs. Rewiring) and Period (Period 1, 2, 3) as within-subject factors were performed separately for the two learning scores (testing old and new knowledge).

#### Testing phase

In this phase, participants responded on all trials, including the ones that were No-go trials in the Rewiring phase. Therefore, both previously Go and No-go trials were analyzed here to test how inhibitory control during rewiring affected the old and new knowledge.

First, all trials were categorized as described above, resulting in four trial types (HL, LH, LL or HH). Second, data were grouped according to the tested sequence (Sequence A vs. Sequence B), both containing ten-ten ASRT blocks. Third, for each participant, each sequence, each trial type, and each response type (Go or No-go in the Rewiring phase), median RTs for correct trials were computed (for raw RTs see Fig. S1b in the Supplementary Information). Fourth, learning scores (‘LL minus HL’ and ‘LL minus LH’ for old and new knowledge, respectively) were computed as described above, separately for Sequence A and Sequence B, and separately for the previously Go vs. No-go trials. Finally, repeated-measures ANOVAs with the tested Sequence (Sequence A vs. Sequence B) and Inhibition (Go vs. No-go) as within-subject factors were performed separately for the two learning scores (testing old and new knowledge). This design enabled us to test (i) whether the old and new knowledge coexisted and was present even when it was irrelevant in a given context (e.g., positive learning score for the old knowledge when tested on Sequence B), and (ii) how inhibitory control during rewiring affected the old and new knowledge in these contexts (by contrasting performance on the previously Go vs. No-go trials, see Fig. 4).

In all analyses, Greenhouse–Geisser epsilon (ε) correction was used when necessary. Original df values and corrected *p* values (if applicable) are reported together with partial eta-squared (η_*p*_^2^) as the measure of effect size. For the significant interactions of the ANOVAs, pair-wise comparisons were performed using LSD post-hoc tests. We report Cohen’s d as a measure of effect size for pair-wise comparisons. Additionally, inverse Bayes factors were computed using default JASP priors (JASP v.0.14.1.0^58^) to see if data provided evidence for the results obtained in the frequentist t-tests (anecdotal evidence for the null-hypothesis: 1 < BF_01_ < 3, at least substantial evidence for the null-hypothesis: BF_01_ > 3; anecdotal evidence for the alternative hypothesis: 1 > BF_01_ > 1/3, at least substantial evidence for the alternative hypothesis: BF_01_ < 1/3)^59^. To provide further contrasts across the learning scores of the old vs. new knowledge, additional analyses were performed where relevant (see Supplementary Information). All statistical tests were two-tailed. Figures were created using the *ggplot2* package^60^.

Although RTs were the primary measures of interest in the current study, we performed similar analyses on the accuracy measures as well. These results are reported in the Supplementary Information, along with the results of the two additional tasks (free generation and triplet sorting tasks), which tested whether participants gained consciously accessible knowledge about the sequence and/or probability structure of the learning task.

## Supplementary Information

Supplementary Information.

## Data Availability

Data used for the analyses reported in this paper are available on the following online repository: ﻿https://osf.io/dt9b8/?view_only=5b6b8850ab8e412a9588a5842870346e.
