# Supplementary material for: Inhibitory control hinders habit change
Source: Sci Rep. 2022 May 18;12:8338. doi: 10.1038/s41598-022-11971-6 (PMC9117234; doi:10.1038/s41598-022-11971-6)
Supplement: Supplementary file 1 — Supplementary Information. [file 41598_2022_11971_MOESM1_ESM.pdf]

**Supplementary information for the manuscript entitled**

**'Inhibitory control hinders habit change'**

Authors: Kata Horváth, Dezso Nemeth, Karolina Janacsek

**Table of contents**

Supplementary introduction: Behavioral and neural characteristics of habit learning across human and animal studies ........ 2

Supplementary results: Reaction times ........ 8

Raw reaction time (RT) performance ........ 8

Figure S1. Raw RT performance throughout the experiment. ........ 8

How does acquisition of new knowledge compare with the initial learning process? ........ 9

Is the level of the new knowledge comparable to that of the old knowledge in the Testing phase? ........ 9

Supplementary results: Accuracy ........ 11

Figure S2. Performance in the Learning and Rewiring phases as measured by accuracy and sensitivity index. ........ 12

Results of the Learning and Rewiring phases. ........ 12

Results of the Testing phase. ........ 13

Figure S3. Performance in the Testing phase as measured by accuracy ........ 15

Supplementary results: Was the acquired knowledge consciously accessible? ........ 16

Free generation task ........ 16

Task and procedure. ........ 16

Statistical analysis. ........ 16

Results. ........ 17

Triplet sorting task ........ 17

Task and procedure. ........ 17

Statistical analysis. ........ 18

Results. ........ 18

Supplementary methods ........ 20

Estimation of required sample size ........ 20

Task and procedure ........ 21

Learning phase. ........ 21

Figure S4. Stimulus- and probability-structure of the task. ........ 23

Rewiring phase. ........ 24

Testing phase. ........ 26

Supplementary references ........ 28

**Supplementary introduction: Behavioral and neural characteristics of habit learning across human and animal studies**

The definition of habits was originally described in animal studies[1,2], highlighting them as behaviors that are elicited by environmental stimuli to which they have become strongly tied and that become insensitive to both outcome (reward) devaluation and contingency degradation[3]. Importantly, it has been recognized that these features and the experimental methods developed to assess habits in animals may not be directly translatable to and likely not sufficient to capture habits in humans[2,4]. Humans are capable of performing tasks without rewards—simply because they are instructed to do so. Therefore, the habitual nature of the acquired associations can be tested using a broader range of methods compared to animals where the outcome devaluation and contingency degradation tests are needed to establish the presence of habitual behaviors[2,5,6]. Moreover, in some cases humans might even use alternative or additional cognitive mechanisms to solve the same task compared to animals (e.g., healthy humans solve a simple concurrent discrimination task using declarative learning processes, while monkeys use habit learning processes[7–9]), further highlighting that different approaches should be favored when testing human habit learning. Indeed, probabilistic classification, sequential decision making, and (motor) sequence learning tasks have all been used to test aspects of habitual behavior in humans as they show similarities with more classical habit learning tasks both on behavioral and neural level[10–15]. (For recent successful attempts at identifying habitual behaviors in more classical habit learning tasks, the outcome devaluation test and the reversal learning task, see[16,17] and the Discussion in the main text.)

Here we present the major similarities—both behavioral and neural—between sequence learning tasks and other commonly used habit learning tasks and argue that despite rarely being employed in the animal literature, sequence learning tasks, including the ASRT used in our study, are valid tools to measure habit learning and change in humans.

On the **behavioral level**, habits in humans are often defined by a collection of attributes that include (i) gradual learning over extended practice; (ii) learning can occur implicitly (i.e., without awareness of what was learned and without conscious control over the acquired knowledge); and (iii) the learned behavior is performed automatically (e.g., without full attention, such as under distraction), even when the behavior becomes no longer relevant (e.g., when environmental contingencies or the outcomes/rewards of the behavior change)[2,18–20]. Notably, it has been recognized that these characteristics of habits do not always cluster together; it is possible that some of these characteristics emerge without others. For example, if

no rewards/reinforcers are given, the other characteristics of habit learning and behavior can still be captured[2,20]. In the next paragraphs, we will discuss how learning and the acquired knowledge in sequence learning tasks and the ASRT, in particular, show many of the defining attributes of habits.

First, habits are learned **gradually, over an extended training period**[4,18]. Learning in SRT-like tasks is likewise gradual and based on extended practice[21]. In the present study, both the Learning phase and the Rewiring phase contained 45 blocks, with 80 stimuli and button presses in each block (excluding the first 5 random trials), that is, the associations were practiced over 3600 trials in each phase. This added up to around an hour and a half of practice per session with breaks between blocks (for more details see the Methods and Supplementary methods sections in the revised MS and SI, respectively). This amount of practice constitutes an extensive training compared to a range of other ASRT studies that focused mainly on earlier phases of learning (with ~1600-2000 trials per session) [e.g., 22–24]. It has been previously shown in healthy human adults that an extended practice of 3600 trials leads to persistent memories of the acquired associations even after a one-year delay that did not include any further practice[25]. Thus, while fewer trials can be sufficient for the initial acquisition of the associations embedded in the task, a more extended practice can help strengthen and automatize the acquired knowledge (see also below). This is why we chose 3600 trials per session in the present study.

Second, habits can be **acquired and performed implicitly,** that is, without awareness of or conscious control over the acquired knowledge[2,4,26]. While learning in deterministic sequence learning tasks often reaches awareness (i.e., participants consciously recognize the repeating sequence) [e.g., 27–29], numerous studies have shown that learning in the ASRT task typically remains fully implicit. The implicit nature of learning and the acquired knowledge can be probed by verbalization, generation, and recognition tests[30]. The verbalization test probes whether participants can verbally declare any task regularities that they may have noticed/learned [e.g., 31,32]. The generation test probes whether participants can consciously control the acquired knowledge by asking them first to generate the regularity present in the task and then generate a new series of responses that do not contain the learned regularities [23,33,34]. In the recognition test, participants are presented with the acquired associations and are asked to decide whether they recognize them or not[15,35]. Based on an extensive list of studies that used verbalization, generation, and/or recognition tests, a recent review conclusively showed that learning and the acquired knowledge remains implicit in the ASRT task [30].

In our study, we used both a generation and a recognition task to test whether participants gained awareness about and conscious control over the acquired knowledge. In the Free generation task, we asked participants to think about the first day then the second day of practice (in a counterbalanced order) and then try to generate the order in which the stimuli appeared. The results showed that they produced the acquired associations at a similar rate for both experimental phases; thus, they did not have conscious control over the acquired knowledge. In the Triplet sorting task, participants were presented with all unique associations (triplets) learned during the Learning phase and the Rewiring phase, separately, and were asked to decide whether the presented associations occurred frequently or not during the given experimental phase. Performance was similarly at chance level for all trial types for both phases (i.e., triplets that were high-probability in the Learning phase but became low-probability in the Rewiring phase, or vice versa, separately in Go and No-go trials), showing that the acquired knowledge was implicit. Further details on these tasks and analyses can be found in the Supplementary results section. We believe that these two measures together with previous studies using the same task and similar samples prove that the acquired associative knowledge in the ASRT task is implicit.

Third, habits are **performed automatically**. For sequence learning tasks, including the ASRT task, a growing body of evidence shows that divided attention[23,36], cognitive load[37], or a secondary task[38] does not affect the learning and expression of associative knowledge. Additionally, using preparatory event-related brain potentials, a recent ASRT study[31] showed that anticipation and processing of stimuli that were predictable based on the acquired associations required less attentional resources compared to unpredictable stimuli; thus, anticipation and processing of the upcoming stimuli were automatic and sensitive to the acquired associative knowledge.

As noted above, in sequence learning tasks, alike most human cognitive tasks, participants provide responses simply because they are instructed to do so. Therefore, the mere fact of responding to a stimulus does not necessarily provide useful information about the automatic stimulus-response (S—R) links that participants learn in these tasks. Instead, automaticity can be assessed using the characteristics of responses such as response speed. For example, in the ASRT task, participants acquire and their responses become driven by probability-based associations between runs of three consecutive stimuli: in some cases, the current stimulus (third of the three) can be predicted with a higher probability based on the two previous stimuli, while in other cases, this predictive probability is low. Once acquired, the first

stimulus automatically elicits the high-probability association when the third stimulus is presented, resulting in faster reaction times than for those third stimuli that are less predictable by the first ones. Thus, S—R links are developed in this task where the speed of the current response is influenced by the combination of the previous and the current stimuli (instead of the current stimulus alone). Indeed, in the present study, participants answered increasingly faster to high-probability stimuli than to low-probability ones, indicating the development of automatic associations as learning progressed.

Further evidence for automaticity in the ASRT task comes from probing whether participants keep responding according to the old associations they have learned even when those associations are no longer relevant. Szegedi-Hallgató et al.[15] used an experimental design similar to ours but without the Go/No-go manipulation: after participants acquired the associations in an extended learning phase of the ASRT task, the sequence was changed in the rewiring phase, and therefore, some of the acquired associations became less relevant (improbable). The authors tested whether errors committed in the rewiring phase were simple motor control errors or so-called anticipatory errors. While a motor control error could be any incorrect response (i.e., pressing any of the three response buttons that are incorrect for a given stimulus), an anticipatory error would reflect the acquired associative knowledge (i.e., pressing the response button that would be an appropriate response for a high-probability triplet even when participants are presented with a low-probability triplet). They found that participants (in the Implicit-Implicit group that is most closely related to our study) committed anticipatory errors based on the old associations they learned in the previous phase, even though those associations were no longer relevant in the rewiring phase.

Another study by Kóbor et al.[32] introduced a pseudorandom environment (i.e., a stimulus stream lacking any regularities) after the initial extended learning phase of ASRT. They found that the associations learned in the initial learning phase were automatically transferred to and influenced participants' responses in the pseudorandom environment, further highlighting the persistence and automaticity of the acquired knowledge. The introduction of a rewiring phase/pseudorandom environment where the initially acquired associations are no longer relevant could be considered as a test to see whether the presented (sequences of) stimuli automatically elicit responses that were appropriate in the old environment. Therefore, although significant differences exist between these experimental designs in humans and the typical tests (with reinforcers) in animal studies, these designs could shed further light on the automatic nature of the acquired associative knowledge in humans. The results of the present study also

reveal the automatic/persistent nature of the acquired knowledge in that the old knowledge persisted in the irrelevant (new) context when probed in the Testing phase.

As discussed so far, evidence shows that sequence learning tasks, and the ASRT task in particular, can be used to test habit learning in humans as the learning process and the acquired associative knowledge exhibit the behavioral characteristics of habits. Further evidence supporting the use of such tasks comes from studies testing the **neural underpinnings of habits**. It has long been recognized that the striatum (a structure within the basal ganglia) plays a key role in habits[39,40]. Specifically, studies have suggested a shift from reliance on the associative striatum (broadly corresponding to the dorsomedial striatum in rodents) during initial learning to the sensorimotor striatum (broadly corresponding to the dorsolateral striatum in rodents) later in learning[4,40–42]. This has often been interpreted as a shift from a goal-directed to a habitual behavior in animal studies[4].

Learning in the (A)SRT task elicits similar brain activation[41,43]. Specifically, a recent meta-analysis of human functional fMRI data revealed converging activation in the striatum, including the anterior segment of the caudate nucleus and the putamen[41], although this study did not contrast early vs. later phases of learning to test the shift in reliance from the associative to the sensorimotor striatum. In another meta-analysis of functional fMRI data, Lohse et al.[43] tested neural increases and decreases over short, medium and long timescales of a range of tasks that included sequence learning tasks as well. They found increased activation in the putamen over the medium and long timescales, suggesting that an increasing automatization on the behavioral level over extended practice is associated with increased involvement of the putamen (sensorimotor striatum). Finally, another meta-analysis contrasted brain activation during different habit learning tasks directly to test whether the human putamen plays a similar role to the rodent dorsolateral striatum in habitual behavior[13]. They found that outcome devaluation, sequential decision-making, and sequence learning tasks likewise elicited activation in the putamen, suggesting that despite being highly different tasks, they rely on similar learning mechanisms. To sum up, converging evidence from brain imaging studies shows that classic habit learning tasks and implicit sequence learning tasks rely on the same basal-ganglia-based network, both in humans and animals.

**In conclusion**, we discussed that, compared to habit research in animals, a broader set of behavioral characteristics are used to capture habits in humans. We provided evidence both from previous research and the current study that these characteristics are present in sequence learning tasks, including the ASRT task. Research probing the neural underpinnings of habits

also revealed that the same basal-ganglia-based network is involved in sequence learning and other, more traditional habit learning tasks.

## Supplementary results: Reaction times

### Raw reaction time (RT) performance

To illustrate how general performance changed throughout the experiment, we present raw RTs in Figure S1.

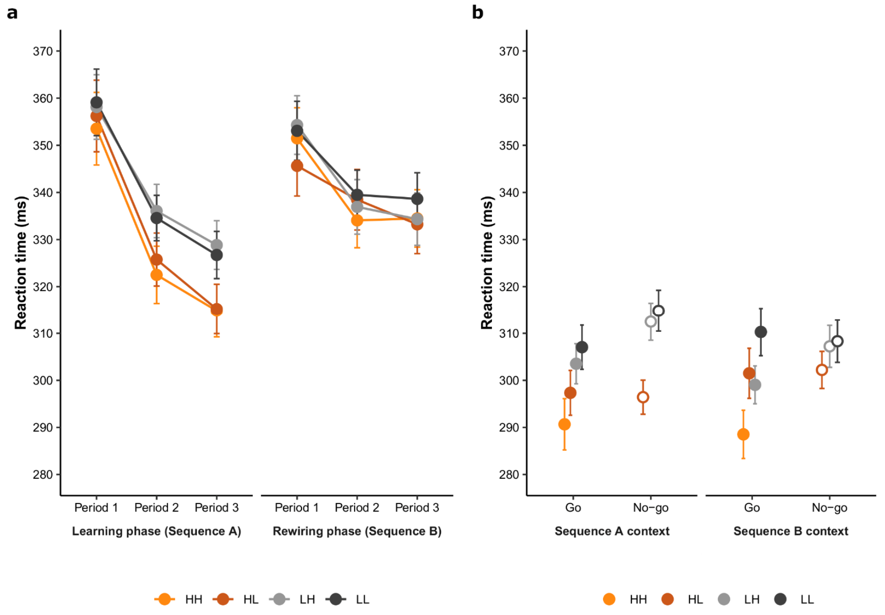


**Figure S1. Raw RT performance throughout the experiment.** a) Mean RTs measured in the Learning and Rewiring phases, separately for the four trial types (HH, HL, LH, LL). During the Learning phase, RTs were faster for the high-probability trial types (HL, HH) compared with the low-probability ones (LH, LL), indicating acquisition of the associations of Sequence A. In the Rewiring phase, participants showed increasingly faster RTs on the LH trials compared to the LL trials, indicating the acquisition of the Sequence B associations (for detailed analyses, see main text). In these two phases, only responses on Go trials are displayed. b) Mean RTs during the Testing phase, separately for four trial types (HH, HL, LH, LL), the previously Go and No-go trials, and the tested contexts (Sequence A vs. B). Please note that there were no No-go trials within the HH trial type, and therefore the primary measures of interest were derived from the other three trial types (for details see Methods section in the main text). When tested on Sequence A, participants expressed the old knowledge both on the Go and No-go trials (faster RTs for HL than for LL). When tested on Sequence B, the new knowledge was present on the Go (faster RTs for LH than for LL trials) but not on the No-go trials; additionally, the old knowledge (associations of Sequence A) also persisted (faster RTs on HL than on LL) even though it was not relevant in this testing context. The interpretation presented here is supported by analyses on the learning scores reported in the main text. Error bars represent the Standard Error of the Mean (SEM).

**How does acquisition of new knowledge compare with the initial learning process?**

To answer this question, we directly compared the acquisition of old knowledge in the Learning phase and the acquisition of new knowledge in the Rewiring phase (measured by the 'LL minus HL' and 'LL minus LH' learning scores, respectively). There was no significant difference between the learning trajectories of the old and new knowledge (Phase x Period interaction: $F(1, 30) = 0.657$, $p = .522$, $\eta_p^2 = .021$; circled areas of Figure 3ab), however, the overall magnitude of learning was greater in the Learning than in the Rewiring phase (7.7 ms and 1.9 ms, respectively; significant main effect of Phase: $F(1, 30) = 6.215$, $p = .018$, $\eta_p^2 = .172$). Learning scores gradually increased, irrespective of the phase (main effect of Period: $F(2, 60) = 7.646$, $p = .001$, $\eta_p^2 = .203$). Overall, these results suggest that, although participants were able to acquire the new knowledge, this process was less successful than the initial acquisition of the old knowledge.

**Is the level of the new knowledge comparable to that of the old knowledge in the Testing phase?**

To test this question, first we performed paired samples t-tests contrasting the old knowledge with the new one in their relevant contexts of the Testing phase (i.e., tested on Sequence A vs. Sequence B, respectively). These data are displayed in the circled areas of Figure 4ab of the main text.

On the Go trials, there was no significant difference between the learning scores ($t(30) = -0.47$, $p = .643$, Cohen's $d = 0.08$, $BF_{01} = 4.715$), suggesting that participants could express both the old and new knowledge in their respective relevant contexts to a similar extent. This finding could be interpreted as flexibility of the acquired knowledge. On the No-go trials, the learning score for the old knowledge was significantly greater than that for the new knowledge ($t(30) = 5.79$, $p < .001$, Cohen's $d = 1.04$ , $BF_{01} = 2.841^{e-5}$). Consistent with the finding that only the learning score for the old knowledge was significantly above zero (reported in the main text), this result reveals the detrimental effect of inhibition: on the previously inhibited trials, the old knowledge was reinstated, and the new knowledge was not expressed (and presumably not acquired).

Next, we performed similar analyses to contrast the level of old and new knowledge in the new (Sequence B) context. Thus, data displayed in the non-circled area of Figure 4a and the circled area of Figure 4b of the main text are contrasted in this analysis. On the Go trials, there

was no significant difference between the learning scores ($p$ = .402, Cohen's $d$ = 0.15; $BF_{01}$ = 3.743, indicating strong evidence for no difference), suggesting that participants could express both the old and new knowledge in the new context to a similar extent. On the No-go trials, the learning score for the old knowledge was significantly greater than that for the new knowledge ($p$ < .050, Cohen's $d$ = 0.37, $BF_{01}$ = 0.849). Again, consistent with the finding that only the learning score for the old knowledge was significantly above zero (reported in the main text), this result further supports the detrimental effect of inhibition.

## Supplementary results: Accuracy

As it is shown in Figure S2a, there was a ceiling effect in accuracy (97.4% on average) in the Rewiring phase, likely due to the introduction of the Go/No-go manipulation in this phase. Therefore, accuracy data of the Rewiring phase and of the Learning phase for comparability, were analyzed as follows: First, we compared average accuracy measured on the Go trials for the four trial types, separately for the two phases using repeated measures ANOVAs with Trial type (HH, LL, LH, HL) and Period (1, 2, 3) as within-subject factors. Next, we calculated sensitivity indices for the Rewiring Phase only, separately for the LL, LH and HL trial types. For these indices, false alarm rate (on No-go trials) was extracted from the ratio of correct responses (on Go trials). Since all HH trials were Go, these trials could not be included in this analysis. The sensitivity indices were submitted to a repeated measures ANOVA with Trial type (LL, LH, HL) and Period (1, 2, 3) as within-subject factors.

Since participants responded on all trials during the Testing phase, accuracy did not show ceiling effect (91% on average, see Figure S2c), and ANOVAs on learning scores could be performed in accordance with the RT analysis reported in the main text. Learning scores were calculated as follows: for old knowledge, accuracy on LL trials were subtracted from accuracy on HL trials; for new knowledge, accuracy on LL trials were subtracted from accuracy on LH trials. In both cases, higher learning scores indicated better knowledge. Repeated measures ANOVAs with the tested Sequence (Sequence A vs. Sequence B) and Inhibition (Go vs. No-go) as within-subject factors were performed separately for the two learning scores (testing old and new knowledge). Additionally, for comparability with RT analyses, we performed one-sample t-tests to reveal whether the learning scores were significantly above zero.

Greenhouse-Geisser epsilon (ε) correction was used when necessary. Original df values and corrected *p* values (if applicable) are reported together with partial eta-squared ($\eta_p^2$) as the measure of effect size. LSD correction was used for pair-wise comparisons to correct for Type I error. We report Cohen's d as a measure of effect size for pairwise comparisons. Additionally, Bayes factors were computed using default JASP priors to see if data provided strong evidence for the results obtained in the frequentist t-tests (anecdotal evidence for the null-hypothesis: $1 < BF_{01} < 3$, at least substantial evidence for the null-hypothesis: $BF_{01} > 3$; anecdotal evidence for the alternative hypothesis: $1 > BF_{01} > 1/3$, at least substantial evidence for the alternative hypothesis: $BF_{01} < 1/3$) [44,45].

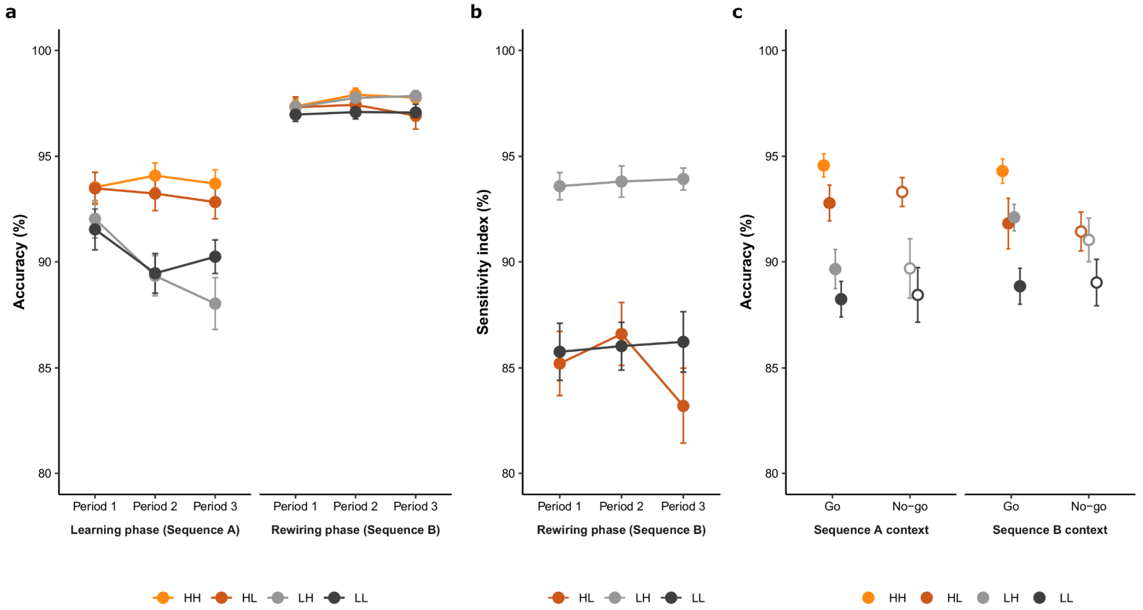


**Figure S2. Performance in the Learning and Rewiring phases as measured by accuracy and sensitivity index.** (a) The analysis of the Learning phase revealed that participants successfully acquired the associations of Sequence A as there were more accurate on those trials that were high-probability compared to those that were low-probability in this phase (HH and HL vs. LL and LH; underlined letters indicating probabilities of the current comparison; see also Figure 2 in main text). Accuracy in the Rewiring phase was very high due to the introduction of No-go trials, and therefore no significant effects could be detected in the analysis. (b) To track rewiring despite the very high accuracy, sensitivity index (ratio of correct responses minus false alarm rate) was computed for the Rewiring Phase. The analysis of this index revealed that participants successfully acquired the associations of Sequence B as the index was higher (i.e., fewer false alarms) for these trials that became high-probability compared to those that were and/or became low-probability in the Rewiring phase (LH vs. LL, and LH vs. HL, respectively). Additionally, participants showed similar sensitivity index for LL and HL trials, suggesting unlearning of the associations of Sequence A during rewiring. (c) During the Testing phase, participants responded to all trials, and old and new knowledge were tested in both the old (Sequence A) and new (Sequence B) contexts. When tested on Sequence A, participants expressed the old knowledge both on the Go and No-go trials (higher accuracy for HL than for LL). When tested on Sequence B, the new knowledge was also present both on the Go and No-go trials (higher accuracy for LH than for LL trials). Additionally, the old knowledge (associations of Sequence A) also persisted (higher accuracy on HL than on LL) even though it was not relevant in this testing context. The interpretation presented here is supported by analyses on the learning scores reported in the "Results of the Testing phase" section below. Error bars represent the SEM.

**Results of the Learning and Rewiring phases.** The analysis of the Learning phase revealed that the associations of Sequence A were successfully acquired. Participants were more accurate on those trials that were high-probability in this phase (that is, HH and HL) than on those that were low-probability (LL and LH) (significant main effect of Trial type: $F(3, 90) = 18.956$, $p$

< .001, $\eta_p^2$ = .387), and this difference increased as the task progressed (significant Period x Trial type interaction: $F$(6, 180) = 5.718, $p$ < .001, $\eta_p^2$ = .160; Figure S2a). This result suggests that participants successfully acquired the associations of Sequence A. The main effect of Period was also significant ($F$(6, 180) = 8.734, ε = .771, $p$ = .002, $\eta_p^2$ = .225) due to the decreasing accuracy on the low-probability trials.

In the analysis of the Rewiring phase, neither the main effects nor the interaction reached significance (main effect of Period: $F$(6, 180) = 0.799, $p$ = .455, $\eta_p^2$ = .026; main effect of Trial type: $F$(3, 90) =2.002, $p$ = .119, $\eta_p^2$ = .063; Period x Trial type interaction: $F$(6, 180) = 0.571, $p$ = .753, $\eta_p^2$ = .019; Figure S2b), likely due to the very high accuracy on all trial types (97.4% on average).

To track rewiring despite the very high accuracy, we performed an ANOVA using the sensitivity index as described above. The ANOVA revealed a significant main effect of Trial type ($F$(2, 60) = 44.178, $p$ < .001, $\eta_p^2$ = .596): the sensitivity index was higher for those trials that became high-probability compared to those that were low-probability throughout the experiment (L$\underline{\text{H}}$ vs. L$\underline{\text{L}}$: $p$ < .001, Cohen's $d$ = 1.66, $BF_{01}$ = $2.471^{e-8}$) as well as compared to those trials that became low-probability in the Rewiring phase only (L$\underline{\text{H}}$ vs. H$\underline{\text{L}}$: $p$ < .001, Cohen's $d$ = 1.45, $BF_{01}$ = $4.415^{e-7}$). This indicates that participants acquired the new knowledge (associations of Sequence B) during the Rewiring phase. At the same time, the latter finding also suggests that unlearning of the old knowledge took place, at least partly, since if participants had responded according to the probabilities of the Learning phase, an opposite pattern would have been expected with higher sensitivity index for the HL than for the LH trials. Moreover, the sensitivity index on those trials that became low-probability only in the Rewiring phase did not differ significantly from that on the trials that were low-probability throughout the experiment (H$\underline{\text{L}}$ vs. L$\underline{\text{L}}$: $p$ = .378, Cohen's $d$ = 0.16, $BF_{01}$ = 3.615), further suggesting unlearning of the old knowledge (associations of Sequence A). . The main effect of Period and the Period x Trial type interaction did not reach significance ($F$(2, 60) = 1, 400, $p$ = .254, $\eta_p^2$ = .045, $F$(4, 120) = 2.345, $p$ = .059, $\eta_p^2$ = .072, respectively).

Altogether, these results are consistent with those of RT measures reported in the main text: namely, the associations of Sequence A and Sequence B were both successfully acquired in the Learning and Rewiring phases, respectively, and the associations of Sequence A seemed to be unlearned during the Rewiring phase.

**Results of the Testing phase.** The ANOVA on the 'HL minus LL' learning score measuring the *old knowledge* (Figure S3a) revealed a significant main effect of Sequence ($F$(1, 30) = 6.095,

$p = .129$, $\eta_p^2 = .169$), with overall smaller learning scores when tested on Sequence B than on Sequence A, suggesting an effect of rewiring. Nevertheless, participants performed above zero in both contexts ($p < .001$, Cohen's $d = 1.18$ and $p = .006$, Cohen's $d = 0.53$, for Sequence A and Sequence B, respectively), suggesting the persistence of the old knowledge both in the relevant (Sequence A) and irrelevant (Sequence B) testing contexts. The main effect of Inhibition ($F(1, 30) = 0.011$, $p = .915$, $\eta_p^2 < .001$) and the Sequence x Inhibition interaction did not reach significance ($F(1, 30) = 0.277$, $p = .603$, $\eta_p^2 = .009$).

In the ANOVA on the 'LH minus LL' learning score measuring the *new knowledge* (Figure S3b), neither of the main effects nor the interaction reached significance (main effect of Sequence: $F(1, 30) = 2.442$, $p = .129$, $\eta_p^2 = .075$; main effect of Inhibition: $F(1, 30) = 0.435$, $p = .514$, $\eta_p^2 = .014$; Sequence x Inhibition interaction: $F(1, 30) = 0.253$, $p = .619$, $\eta_p^2 = .008$). Nevertheless, for comparability with RT analyses, we performed one-sample t-tests to reveal whether any of the learning scores of the new knowledge were significantly above zero. These t-tests revealed that learning scores in the new (Sequence B) testing context were significantly above zero both on the previously Go ($p < .001$, Cohen's $d = 0.86$, $BF_{01} = 0.002$) and No-go trials ($p = .043$, Cohen's $d = 0.38$, $BF_{01} = 0.756$). Opposingly, in the old (Sequence A) testing context, learning scores seemed to be at zero-level both for the No-go trials ($p = .397$, Cohen's $d = 0.15$, $BF_{01} = 3.717$) and Go trials ($p = .158$, Cohen's $d = 0.26$, $BF_{01} = 2.030$).

Overall, the accuracy results of the Testing phase suggest the persistence of old knowledge both in the relevant (Sequence A) and irrelevant (Sequence B) testing contexts, and the simultaneous presence of the new knowledge that was expressed in its relevant (Sequence B) testing context but not in the old (Sequence A) testing context.

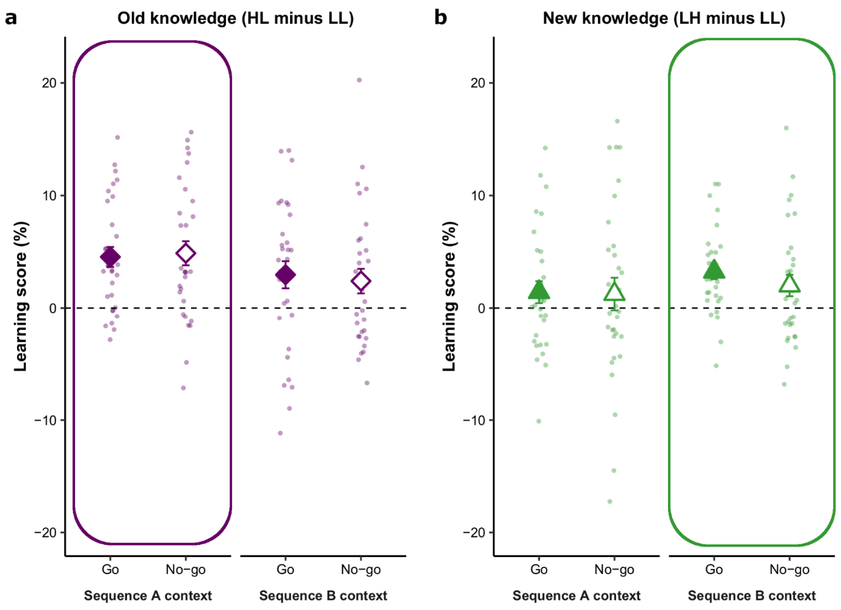


**Figure S3. Performance in the Testing phase as measured by accuracy.** (a) The analysis of the ‘HL minus LL’ learning score revealed that the old knowledge was expressed both when tested in the old, relevant context (Sequence A) and in the new context (Sequence B), in which this knowledge was irrelevant. Inhibition of responses during rewiring did not significantly affect these results. (b) The analysis of the ‘LH minus LL’ learning score revealed that the new knowledge was expressed in the new (Sequence B) context, in which it was relevant, but not in the other context. Again, inhibition of responses during rewiring did not significantly affect these results. Error bars represent the SEM.

## Supplementary results: Was the acquired knowledge consciously accessible?

At the end of the Testing phase, a free generation task[15,46] and a triplet sorting task[15,47,48] were administered to probe whether participants acquired consciously accessible knowledge about the probability structure of the task using recall- and recognition-based approaches, respectively.

### Free generation task

**Task and procedure.** In this task, participants were asked to generate a series of responses that followed the order within which stimuli appeared in the ASRT task. The task was administered for Sequence A and Sequence B separately by asking participants to remember what they practiced on Day 1 and on Day 2, respectively, and generate series of responses similar to those that they practiced[46]. This task was used to test if participants could consciously access and control their old and/or new knowledge (associations of Sequence A and B, respectively) to generate responses according to the testing conditions[27,46,49]. The two conditions (Sequence A and B) were administered in a counterbalanced order and each consisted of four runs with 24 button presses. Participants were asked to use the same response buttons as in the ASRT task.

**Statistical analysis.** To test the performance on the free generation task, first, trials (responses) were categorized as being high- or low-probability both according to Sequence A and Sequence B. This resulted in four trial types (HH, HL, LL, LH), similar to the analyses of the ASRT task. Since three consecutive trials were needed to identify the third trial's probability, 22 trials were evaluated in each run. Second, responses that corresponded to HH or LL triplets were excluded from the analyses because these were the same in Sequence A and B and, therefore, could not be used to probe if participants gained conscious knowledge separately about Sequence A (old knowledge) or Sequence B (new knowledge). Third, the percentage of HL and LH responses out of all evaluated ones (22) were calculated for each run, both for Sequence A and Sequence B. Fourth, we computed averages of these percentages across the four runs in each condition.

HL responses could be interpreted as the knowledge of Sequence A since these reflect high-probability triplets in Sequence A, and LH responses could be interpreted as the knowledge of Sequence B since these reflect high-probability triplets in Sequence B. Therefore, if participants generated more HL than LH responses in the Sequence A condition and/or more LH than HL responses in the Sequence B condition, that would indicate that they gained

consciously accessible knowledge that they could use to control their responses according to the testing conditions[46]. To test this possibility, the percentages of HL vs. LH responses were compared using paired samples t-tests, separately in Sequence A and Sequence B conditions. Additionally, Bayes factors were computed using default JASP priors for all pairwise comparisons[2].

**Results.** The percentages of HL and LH responses did not differ significantly either in the Sequence A ($M_{HL}$ = 23.70% vs. $M_{LH}$ = 21.20%; $t(30) = 1.176$, $p = .249$, Cohen's $d = 0.21$, $BF_{01} = 5.138$) or Sequence B conditions ($M_{HL}$ = 22.27% vs. $M_{LH}$ = 21.46%; $t(30) = 0.456$, $p = .652$, Cohen's $d = 0.08$, $BF_{01} = 4.210$). This indicates that participants could not consciously control their old or new knowledge to generate their responses according to the testing conditions.

Overall, the results of the free generation task indicate that participants did not gain consciously accessible knowledge either about the associations of Sequence A (old knowledge, tested by HL responses) or Sequence B (new knowledge, tested by LH responses) that they could use to generate their responses according to the testing conditions, thus they remained implicit.

### Triplet sorting task

**Task and procedure.** In this task, participants saw each unique triplet of the practiced sequences and were asked to make forced-choice decisions on their occurrence probability to probe if they gained any consciously accessible knowledge about the learned/rewired associations. The same stimuli were used as in the ASRT task: that is, participants saw a picture with the dog's head appearing in one of the four possible stimulus locations. The three consecutive stimuli that formed a triplet were presented as follows: the first one was presented for 700 ms on the upper third of the screen, then the second one was added to the middle of the screen for another 700 ms. Finally, the third one was also added to the lower third of the screen. All three stimuli remained on the screen until a response was provided. Participants were instructed to remember what they practiced on Day 1 and Day 2, respectively, and decide whether the presented triplet occurred frequently (high-probability triplets) or not (low-probability triplets) for the particular experimental phase (Day 1 vs. Day 2). Thus, all 64 unique triplets were presented to the participants twice: once for determining their probabilities on Day 1 (i.e., in Sequence A) and once for determining their probabilities on Day 2 (i.e., in Sequence B), in a counterbalanced order.

**Statistical analysis.** To analyze the performance on the triplet sorting task, first we determined correct responses as identifying high-probability triplets as high-probability and low-probability triplets as low-probability separately in the two conditions of the task (i.e., when tested on Sequence A and on Sequence B). Next, we calculated the percentage of correct responses for all four trial types (HH, LL, LH, HL), for both conditions. As in the free generation task, the HL and LH categories were the primary measures of interest. If participants had more correct responses on HL trials in Sequence A than in Sequence B, or vice versa, more correct responses on LH trials in Sequence B than in Sequence A, that would indicate that they gained consciously accessible knowledge about the associations and could successfully identify in which phase of the experiment those associations were more probable.

Since all unique triplets were presented in this task, it enabled us to also categorize triplets based on whether they were inhibited during rewiring or not (i.e., No-go vs. Go trials, respectively) and test the percentage of correct responses as a function of inhibition as well. This resulted in eight measures that were included in the analysis: the percentage of correct responses for HL and LH trials, separately for the previously No-go and Go trials, and separately for Sequence A and Sequence B.

First, we conducted a repeated measures ANOVA on the percentage of correct responses with Trial type (LH vs. HL), Inhibition (Go vs. No-go), and Sequence (tested on Sequence A vs. on Sequence B) as within-subject factors to probe if any of these factors or their interactions affected participants' responses. Finally, all eight measures were tested against chance level (50%) using one-sample t-tests, supplemented by Bayes factors using default JASP priors[2].

**Results.** The frequentist ANOVA did not reveal any significant main effects or interactions (all *p*s > .221, all $\eta_p^2 < .05$), suggesting that participants' decisions did not differ as a function of trial type (HL vs. LH), whether they suppressed responses to the tested trials during rewiring (No-go vs. Go), or whether they were tested on Sequence A or on Sequence B.

The percentage of correct responses (ranging from 47.7% to 55.5%) did not differ significantly from chance level (50%) for any of the eight measures (all *p*s ≥ .280, Cohen's *d*s ≤ 0.20, $BF_{01}$s ≥ 3.544 with the exception of LH No-go trials tested on Sequence B where $BF_{01}$ = 2.547), suggesting that participants categorized the triplets as high- or low-probability randomly.

Overall, these results indicate that participants did not gain consciously accessible knowledge about the practiced associations in the experiment, irrespective of the tested

knowledge, inhibition, and testing condition, further reflecting that the acquired knowledge remained implicit.

## Supplementary methods

### Estimation of required sample size

We calculated the required sample size based on previously published data obtained from the ASRT task. It has been reported that as few as N = 6-7 participants are sufficient to show a significant learning effect with 25 blocks of ASRT at $p = .05$ and power = .80[50,51]. Since in the current study, we used 45 blocks of ASRT, even a smaller sample is expected to be enough to show a significant learning effect.

Nevertheless, as the main focus of the present study was to investigate the rewiring of the acquired knowledge, we conducted further calculations based on the data of Szegedi-Hallgató et al.[15] (Implicit-Implicit group) obtained in a similar task design but without the Go/No-go manipulation using G*Power 3.1[52]. Based on the mean (13.9 ms) and standard deviation (8.59 ms) of the overall learning score of the new knowledge ('LL minus LH') measured over the 45 blocks of the Rewiring phase, the estimated effect size was Cohen's $d$ = 1.62. The required sample size to show this effect at the level of $\alpha = .05$ and with power = .80 is N = 5.

Next, we calculated learning scores corresponding to the old and new knowledge ('LL minus HL' and 'LL minus LH', respectively) measured in their corresponding context (A and B, respectively) in the Testing phase of the same dataset. Accordingly, to show that the old knowledge ($M$ = 12.6 ms, $SD$ = 16.44 ms, Cohen's $d$ = 0.77) was expressed (i.e., was significantly above zero) after rewiring, a sample of N = 12 is needed at $p = .05$ and power = .80. In a similar calculation, we found that to show that the new knowledge ($M$ = 18.2 ms, $SD$ = 17.18 ms, Cohen's $d$ = 1.06) was successfully exhibited in its context, a sample size of N = 8 is required. Importantly, these estimates need to be treated with caution due to the task lacking the Go/No-go manipulation. Consequently, we performed the required sample size analysis with a more stringent criterion as well, expecting a medium effect size (Cohen's $d$ = 0.50) both for the old and new knowledge, with at $p = .05$ and power = .80. This calculation revealed that a sample with N = 27 is required to show significant effects for the old and new knowledge in the Testing phase. Thus, the sample size of our study meets the estimated criteria, including the more stringent one.

Since there is no previous ASRT study with a Go vs. No-go manipulation, for direct comparison of performance on the Go vs. No-go trials within a given context or performance

on Go/No-go trials across contexts, we could not use estimates from previously published studies. However, using the same criteria as above (i.e., expecting a medium effect size of 0.5 with $p$ = .05 and power = .80) we found that N = 27 participants are required to show significantly better performance, for example, on the Go vs. the No-go trials (i.e., one-tailed paired-samples comparison), while allowing significant deviation in either direction (e.g., better performance on Go or on No-go trials, two-tailed comparison) resulted in a required sample size of N = 34. Thus, overall, based on our calculations, a sample size of N = 27-34 would be sufficient for our study. During the recruitment process, we managed to collect data of 33 participants, and the final sample consisted of 31 participants (see Participants section in the main text).

Finally, there are cases where near-zero performance could be expected (e.g., expressing old knowledge in the new context, or vice versa, expressing new knowledge in the old context). Calculating required sample size for these cases would be inappropriate as they would emerge as non-significant results during the analysis. For such non-significant results, Bayes factors could be used to see if there is sufficient evidence for the null-hypothesis (i.e., no difference from zero/non-significant result). At the same time, Bayes factors could also reveal if there is sufficient evidence in the data for the alternative hypothesis. These calculations could be used to confirm/provide further support for the interpretations of significant results. Therefore, we reported Bayes factors where appropriate both for non-significant and significant pair-wise comparisons. For more details on Bayes factors, see the Statistical analysis section in the main text.

## Task and procedure

**Learning phase.** In the ASRT task[22,53], the target stimulus (a dog's head) appeared in one of the four horizontally arranged circles on the screen (see Figure 1 in the main text). Four buttons of a response box (Chronos, Psychology Software Tools) corresponded to the four locations. Participants were asked to press the corresponding button when the stimulus appeared on the screen as fast and as accurately as they could. Unbeknownst to the participants, the stimulus presentation order followed an eight-element sequence, in which predetermined pattern (P) trials alternated with random ones (e.g., 1 – r – 3 – r – 4 – r – 2 – r, where numbers denote the four predetermined locations on the screen from left to right, and *r*s denote the randomly chosen locations out of the possible four).

The task was organized into blocks. One block consisted of 85 trials. In the first five trials, randomly chosen locations were presented and served as a warm-up; this was followed by ten repetitions of the eight-element alternating sequence. Stimuli were presented on the screen until the correct response was provided, followed by a screen with the four empty circles for 120 ms (response-to-stimulus interval, RSI). After each block, participants received feedback about their average RT and accuracy presented for five seconds. If the average accuracy was lower than 80% (irrespective of the average RT), participants were instructed to answer more accurately. If the average accuracy was higher than 95% and the average RT was slower than 250 ms, participants were instructed to answer faster. These settings ensured a good balance between speed and accuracy while encouraging fast responses characteristic of automatic skills and habits[54,55]. After the feedback, a short self-paced break was administered before the next block started. Overall, the Learning phase consisted of 45 blocks (around 45 min), divided into three periods of 15 blocks with five-min breaks in-between to reduce potential fatigue effects[56]. Thus, participants completed 450 repetitions of the sequence (3600 trials, excluding the warm-up trials) in this phase. This extensive practice ensured the acquisition of sound knowledge of the stimulus regularities that has been shown to persist even after a one-year delay (without any further practice)[24,25] and that could serve as a good experimental model for learning processes underlying automatic skills and habits[15,24].

Due to the alternating sequence of stimulus presentation, some runs of three consecutive trials (triplets) were more probable than others. For instance, in the 1 – r – 3 – r – 4 – r – 2 – r sequence, the 1 – x – 3, 3 – x – 4, 4 – x – 2, and 2 – x – 1 triplets (where x denotes the middle element of the triplet) occurred with a greater probability because they were presented in every sequence repetition and could also be formed by chance (see Figure S4). (Notably, since participants were unaware of the alternating regularity, triplets with identical stimuli but with the third element in different (P or r) position were indistinguishable to them[22,53], and responses to them were therefore combined in the analyses.) Meanwhile, for instance, triplets 1 – x – 2 and 4 – x – 3 occurred with a lower probability since they could only be formed by chance. The former triplets are referred to as high-probability triplets, while the latter ones are referred to as low-probability triplets. For all triplets, the third element ($n$) of a triplet was predictable by the first one ($n$-2) of that triplet with a higher or lower probability, while the middle element ($n$-1) did not have a predictive value. Triplets that had the same first and third elements but different middle elements (e.g., 1 – 1 – 3, 1 – 2 – 3, 1 – 3 – 3, and 1 – 4 – 3 for the triplet 1 – x – 3) were therefore treated as identical in all analyses. Importantly, triplets were identified using a moving window throughout the stimulus stream. Thus, *each trial* was categorized as the last element of

a high- or low-probability triplet, and this categorization was used for the RT and accuracy analyses; the same trial then served as the middle and the first element for the categorization of the following triplets.

There were 64 unique triplets in the task, including all pattern-ending (50%) and random-ending (50%) triplets. Sixteen of these unique triplets were of high-probability and 48 triplets were of low-probability. Since high-probability triplets could occur as pattern-ending triplets (50% of all trials) and by chance as random-ending triplets (12.5% of all trials), these triplets constituted 62.5% of all trials in a given session (Figure S4b). Low-probability triplets constituted the remaining 37.5% of the trials; these were all random-ending triplets. Consequently, on the level of unique triplets, high-probability triplets were five times more probable than the low-probability ones (approx. 4% [62.5% / 16] vs. 0.8% [37.5% / 48]). Note that within the low-probability triplets, trills (that is, triplets with the same stimulus as the first and third elements, such as 1 – x – 1), including repetitions (such as 1 – 1 – 1), were excluded from all analyses because participants typically show preexisting response tendencies to them[24,25].

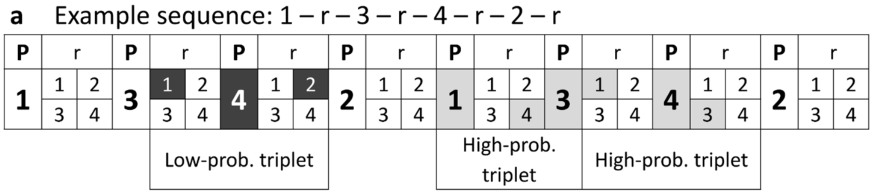


b

| | Structure: P – r – P<br>e.g., 1 – r – 3 | Structure: r – P – r<br>e.g., r – 4 – r |
|---|---|---|
| **High-probability triplets**<br>(62.5% of all trials) | e.g., 1 – 4 – 3 (50%) | e.g., 1 – 4 – 3 (12.5%) |
| **Low-probability triplets**<br>(37.5% of all trials) | never occurring<br>(always high) | 1 – 4 – 1 (12.5%)<br>1 – 4 – 2 (12.5%)<br>1 – 4 – 4 (12.5%) |

**Figure S4. Stimulus- and probability-structure of the task.** (a) Stimulus presentation order followed an eight-element sequence in which predetermined pattern (P) trials alternated with random (r) ones. In the example sequence on the figure, numbers correspond to the four possible locations on the screen from left to right, and the *r*s denote the randomly chosen locations out of the possible four. Due to the alternating sequence structure, some runs of three successive trials (triplets) occurred with a higher probability (light grey) than others (dark grey). These are referred to as high- and low-probability triplets, respectively. For all triplets, the third element (*n*) of a triplet was predictable by the first element (*n*-2) of that triplet with a higher or lower probability, while the middle element (*n*-1) did not have a predictive value. Importantly, triplets were identified using a moving window

throughout the stimulus stream. Thus, *each trial* was categorized as the last element of a high- or low-probability triplet, and this categorization was used for the RT and accuracy analyses; the same trial then served as the middle and the first element for the categorization of the following triplets. (b) Since high-probability triplets could occur as pattern-ending triplets (P – r – P structure; 50% of all trials in a given task session) and by chance as random-ending triplets (r – P – r structure; 12.5% of the trials), these triplets constituted 62.5% of all trials. Low-probability triplets constituted the remaining 37.5% of the trials; these were all random-ending triplets. On the level of unique triplets, high-probability triplets were five times more probable than low-probability triplets.

**Rewiring phase.** In this phase, a structural change was introduced to the task by replacing Sequence A with Sequence B to prompt the rewiring of old knowledge[15]. Additionally, participants were allowed to respond on some trials (Go trials) but were asked to suppress their response on other trials (No-go trials; see Figure 1 in the main text).

For the Go trials, stimulus was presented until the correct response was provided, followed by a 120 ms RSI. For the No-go trials, stimulus was presented for 1000 ms, followed by a 120 ms delay. In case of a false alarm (i.e., when participants made a response on a No-go trial), the stimulus disappeared from the screen and a warning (a red exclamation mark) was presented for 700 ms, followed by the 120 ms delay. After each block, participants received feedback presented for five seconds. If the average accuracy on the Go trials was lower than 80% (irrespective of the average RT), participants were instructed to answer more accurately. If the average accuracy was higher than 95% and the average RT was slower than 400 ms on the Go trials, participants were instructed to answer faster. Additionally, if participants made more than three false alarms on the No-go trials, they were instructed to follow the instructions more carefully and suppress their responses on these trials. These stimulus timing and feedback settings were determined based on pilot data and were used to ensure that inhibitory control processes were engaged during the No-go trials by providing sufficient time for the activation of the automatic response that then had to be suppressed. The fine-tuned feedback provided a good balance between speed and accuracy; specifically, allowing three false alarms encouraged an overall faster response speed (on the Go trials) to promote rewiring. After the feedback, a short self-paced break was administered before the next block started. As in the Learning phase, the task consisted of 45 blocks (around 45 min), divided into three periods of 15 blocks with five-min breaks in-between. Thus, participants completed 450 repetitions of Sequence B (3600 trials, excluding the warm-up trials) in the Rewiring phase.

Due to the introduction of Sequence B in the Rewiring phase, the probability of some triplets changed: 75% of triplets that were high-probability in the Learning phase became low-probability (HL; thus, the first letter refers to the triplet probability in Sequence A, while the

second letter refers to the probability of the same triplet in Sequence B), and they were replaced by new high-probability triplets that were initially low-probability (LH). Meanwhile, occurrence probability of other triplets remained the same: either being low-probability (LL) or high-probability (HH) in both phases. The HL triplets allowed the assessment of initial acquisition and subsequent unlearning of *old knowledge:* participants could acquire that knowledge in the Learning phase and then had to unlearn it in the Rewiring phase when these triplets become low-probability. The LH triplets allowed the assessment of the acquisition of *new knowledge* as part of the rewiring process: as these triplets became high-probability in the Rewiring phase, knowledge about them could be acquired in this phase. The LL triplets served as a baseline to control for general (i.e., probability-independent) practice and/or fatigue effects (for further details on how the learning scores were calculated see Figure 2b and the Statistical analysis section in the main text). The HH triplets were not used in the analyses; these triplets were included in the design only to have largely but not completely different sequences for the Learning and Rewiring phases as explained above.

An example sequence pair used in the Learning and Rewiring phases is shown on Figure 2a. In this example, the 1 – x – 3 triplets (including all four variations with different middle elements, i.e., 1 – 1 – 3, 1 – 2 – 3, 1 – 3 – 3, and 1 – 4 – 3) were high-probability in both phases (HH). Triplets 3 – x – 4, 4 – x – 2, and 2 – x – 1 (12 triplets overall, including all four variations with different middle elements) were initially high-probability but they became low-probability (HL) because they could occur only by chance (i.e., r – P – r structure) in the Rewiring phase. Triplets 3 – x – 2, 4 – x – 1, and 2 – x – 4 triplets (12 triplets overall, including all four variations with different middle elements) that were initially low-probability became high-probability (LH) because they could occur both as part of the predetermined sequence (P – r – P structure) and by chance (r – P – r structure) in the Rewiring phase. The remaining triplets were low-probability in both phases (LL). We chose sequence pairs that were largely but not completely different for the Learning and Rewiring phases because we believe this resembles everyday examples of changing habit-like behaviors more closely. Specifically, when we try to change our routines (e.g., starting to divide household waste into different bins depending on its material), some steps of the routines may remain unchanged (e.g., still collecting non-recyclable items the same way as before), while other steps need rewiring (e.g., putting items made of glass in a different bin).

The assignment of triplets to Go vs. No-go trials was as follows: Two-thirds of HL triplets were No-go trials (e.g., the 4 – x – 2 and 2 – x – 1 triplets in the above example, including all four variations with different middle elements) to promote the use of inhibitory control

during the unlearning of those triplets that were initially high-probability but then became low-probability in this phase. The remaining one-third of HL triplets were Go trials (e.g., the 3 – x – 4 triplets) to allow comparison of performance later in the Testing phase on those trials that were Go vs. No-go in the Rewiring phase. At the same time, two-thirds of LH triplets were Go trials (e.g., the 2 – x – 4 and 4 – x – 1 triplets in the example above) to promote the acquisition of new knowledge by actively responding on those trials that were initially low-probability and became high-probability in the Rewiring phase. The remaining one-third of LH triplets were No-go trials (e.g., the 3 – x – 2 triplets) for the same reason as above. The assignment of different proportions of HL vs. LH triplets to Go and No-go trials aimed to mimic assumptions about how habit-like behaviors would be rewired in everyday situations, that is, by largely inhibiting the old, unwanted behaviors (more No-go trials on HL triplets) and, at the same time, actively engaging in the new, preferred behaviors (more Go trials on LH triplets). Since performance on LL triplets was used as a baseline throughout the experiment, they were also split into Go and No-go trials following a 2:1 ratio. Finally, as the HH category included only one set of triplets (1 – x – 3 in the example above), they were all Go trials. The ratio of Go and No-go trials across triplet categories was about 50:50 (with some variability due to the randomly chosen locations) to control for general expectation biases.

Overall, eight sequence pairs were selected so that the change in triplet probabilities from the Learning to the Rewiring phase followed the details outlined above. These sequence pairs were used in a counterbalanced order to control for any potential idiosyncrasies in how participants may respond to a particular triplet irrespective of its probability. The allocation of triplets to Go and No-go trials was also counterbalanced across sequence pairs and, therefore, participants. This carefully counterbalanced design ensured that the obtained findings were generalizable across sequences and were not due to pre-existing tendencies on one particular sequence pair.

**Testing phase.** In this phase, participants performed 20 blocks of the ASRT task (around 20 min) with the same stimulus timing and feedback settings as in the Learning Phase. Unbeknownst to them, knowledge on both Sequence A and Sequence B was tested. In a counterbalanced order, participants completed five blocks containing one sequence (A or B), then five blocks containing the other sequence (B or A), then the whole procedure was repeated once more, resulting in altogether ten-ten task blocks with each sequence (in ABAB or BABA order; see Figure 1 in the main text). Participants responded on all trials, including the ones that were No-go in the Rewiring phase. This enabled the testing of how inhibitory control affected

(un)learning processes during rewiring. After completing the ASRT task, participants were debriefed and informed that the stimuli followed a predetermined order in the task. Then, a free generation task and a triplet sorting task were administered to probe whether participants acquired consciously accessible knowledge about the sequence and/or probability structure of the task (for details, see section 'Supplementary results: Was the acquired knowledge consciously accessible?').

**Supplementary references**


1. Robbins, T. W. & Costa, R. M. Habits. *Current Biology* **27**, R1200–R1206 (2017).
2. Foerde, K. What are habits and do they depend on the striatum? A view from the study of neuropsychological populations. *Current Opinion in Behavioral Sciences* **20**, 17–24 (2018).
3. Dickinson, A. Actions and habits: the development of behavioural autonomy. *Philosophical Transactions of the Royal Society of London. B, Biological Sciences* **308**, 67–78 (1985).
4. Ashby, F. G., Turner, B. O. & Horvitz, J. C. Cortical and basal ganglia contributions to habit learning and automaticity. *Trends Cogn Sci* **14**, 208–215 (2010).
5. de Wit, S. *et al.* Shifting the balance between goals and habits: Five failures in experimental habit induction. *Journal of Experimental Psychology: General* **147**, 1043 (2018).
6. Du, Y., Krakauer, J. & Haith, A. The relationship between habits and motor skills in humans. *Trends in Cognitive Sciences* **26**, 371–387 (2022).
7. Knowlton, B. J. & Patterson, T. K. Habit Formation and the Striatum. *Current Topics in Behavioral Neurosciences* **37**, 275–295 (2016).
8. Fernandez-Ruiz, J., Wang, J., Aigner, T. G. & Mishkin, M. Visual habit formation in monkeys with neurotoxic lesions of the ventrocaudal neostriatum. *Proceedings of the National Academy of Sciences* **98**, 4196–4201 (2001).
9. Bayley, P. J., Frascino, J. C. & Squire, L. R. Robust habit learning in the absence of awareness and independent of the medial temporal lobe. *Nature 2005 436:7050* **436**, 550–553 (2005).
10. Dezfouli, A. & Balleine, B. W. Habits, action sequences and reinforcement learning. *European Journal of Neuroscience* **35**, 1036–1051 (2012).
11. Doll, B. B., Duncan, K. D., Simon, D. A., Shohamy, D. & Daw, N. D. Model-based choices involve prospective neural activity. *Nature Neuroscience 2015 18:5* **18**, 767–772 (2015).
12. Wood, W. & Rünger, D. Psychology of habit. *Annu Rev Psychol* **67**, 289–314 (2016).
13. Patterson, T. K. & Knowlton, B. J. Subregional specificity in human striatal habit learning: a meta-analytic review of the fMRI literature. *Current Opinion in Behavioral Sciences* **20**, 75–82 (2018).
14. Ambrus, G. G. *et al.* When less is more: Enhanced statistical learning of non-adjacent dependencies after disruption of bilateral DLPFC. *Journal of Memory and Language* **114**, 104144 (2020).
15. Szegedi-Hallgató, E. *et al.* Explicit instructions and consolidation promote rewiring of automatic behaviors in the human mind. *Scientific Reports* **7**, 4365 (2017).

16. Luque, D., Molinero, S., Watson, P., López, F. J. & le Pelley, M. E. Measuring habit formation through goal-directed response switching. *Journal of Experimental Psychology: General* **149**, 1449–1459 (2020).

17. Hardwick, R. M., Forrence, A. D., Krakauer, J. W. & Haith, A. M. Time-dependent competition between goal-directed and habitual response preparation. *Nature Human Behaviour* **3**, 1252–1262 (2019).

18. Ashby, F. G. & Crossley, M. J. Automaticity and multiple memory systems. *Wiley Interdisciplinary Reviews: Cognitive Science* **3**, 363–376 (2012).

19. Schneider, W. & Shiffrin, R. M. Controlled and automatic human information processing: I. Detection, search, and attention. *Psychological Review* **84**, 1–66 (1977).

20. Seger, C. A. & Spiering, B. J. A critical review of habit learning and the basal ganglia. *Frontiers in Systems Neuroscience* **5**, 66 (2011).

21. Henke, K. A model for memory systems based on processing modes rather than consciousness. *Nature Reviews Neuroscience* **11**, 523 (2010).

22. Nemeth, D. *et al.* Sleep has no critical role in implicit motor sequence learning in young and old adults. *Exp Brain Res* **201**, 351–358 (2010).

23. Horváth, K., Török, C., Pesthy, O. & Nemeth, D. Divided attention does not affect the acquisition and consolidation of transitional probabilities. *Scientific Reports* **10**, 1–14 (2020).

24. Romano, J. C., Howard Jr, J. H. & Howard, D. v. One-year retention of general and sequence-specific skills in a probabilistic, serial reaction time task. *Memory* **18**, 427–441 (2010).

25. Kóbor, A., Janacsek, K., Takács, Á. & Nemeth, D. Statistical learning leads to persistent memory: Evidence for one-year consolidation. *Scientific Reports* **7**, 760 (2017).

26. Graybiel, A. M. Habits, Rituals, and the Evaluative Brain. *Annual Review of Neuroscience* **31**, 359–387 (2008).

27. Jimenez, L., Vaquero, J. M. M. & Lupiáñez, J. Qualitative differences between implicit and explicit sequence learning. *Journal of experimental psychology: Learning, Memory, and Cognition* **32**, 475 (2006).

28. Rüsseler, J. & Rösler, F. Implicit and explicit learning of event sequences: evidence for distinct coding of perceptual and motor representations. *Acta Psychologica* **104**, 45–67 (2000).

29. Horváth, K. *et al.* Error Processing During the Online Retrieval of Probabilistic Sequence Knowledge. *Journal of Psychophysiology* **35**, 61–75 (2021).

30. Vékony, T., Ambrus, G. G., Janacsek, K. & Nemeth, D. Cautious or causal? Key implicit sequence learning paradigms should not be overlooked when assessing the role of DLPFC (Commentary on Prutean et al.). *Cortex* **148**, 222–226 (2022).

31. Kóbor, A. *et al.* Implicit anticipation of probabilistic regularities: Larger CNV emerges for unpredictable events. *Neuropsychologia* **156**, 107826 (2021).

32. Kóbor, A., Horváth, K., Kardos, Z., Nemeth, D. & Janacsek, K. Perceiving structure in unstructured stimuli: Implicitly acquired prior knowledge impacts the processing of unpredictable transitional probabilities. *Cognition* **205**, 104413 (2020).

33. Jacoby, L. L. A process dissociation framework: Separating automatic from intentional uses of memory. *J Mem Lang* **30**, 513–541 (1991).

34. Destrebecqz, A. & Cleeremans, A. Can sequence learning be implicit? New evidence with the process dissociation procedure. *Psychon Bull Rev* **8**, 343–350 (2001).

35. Bennett, I. J., Romano, J. C., Howard, J. H. & Howard, D. v. Two Forms of Implicit Learning in Young Adults with Dyslexia. *Ann N Y Acad Sci* **1145**, 184–198 (2008).

36. Jimenez, L. & Mendez, C. Which attention is needed for implicit sequence learning? *Journal of experimental Psychology: learning, Memory, and cognition* **25**, 236 (1999).

37. Horváth, K. *et al. Manipulation of cognitive control does not influence implicit procedural learning - Evidence from a sequential Eriksen-flanker task*. (2022).

38. Nemeth, D. *et al.* Interference between sentence processing and probabilistic implicit sequence learning. *PLoS One* **6**, e17577 (2011).

39. Graybiel, A. M. The Basal Ganglia and Chunking of Action Repertoires. *Neurobiology of Learning and Memory* **70**, 119–136 (1998).

40. Graybiel, A. M. & Grafton, S. T. The striatum: Where skills and habits meet. *Cold Spring Harbor Perspectives in Biology* **7**, (2015).

41. Janacsek, K. *et al.* Sequence learning in the human brain: A functional neuroanatomical meta-analysis of serial reaction time studies. *Neuroimage* **207**, 116387 (2020).

42. Steiner, H. & Tseng, K. Y. *Handbook of basal ganglia structure and function*. (Academic Press, 2016).

43. Lohse, K. R., Wadden, K., Boyd, L. A. & Hodges, N. J. Motor skill acquisition across short and long time scales: A meta-analysis of neuroimaging data. *Neuropsychologia* **59**, 130–141 (2014).

44. Jarosz, A. F. & Wiley, J. What are the odds? A practical guide to computing and reporting Bayes factors. *The Journal of Problem Solving* **7**, 2 (2014).

45. Wagenmakers, E.-J., Wetzels, R., Borsboom, D. & van der Maas, H. L. J. Why psychologists must change the way they analyze their data: the case of psi: comment on Bem (2011). *Journal of Personality and Social Psychology* **100**, 426–432 (2011).

46. Gaillard, V., Vandenberghe, M., Destrebecqz, A. & Cleeremans, A. First-and third-person approaches in implicit learning research. *Consciousness and Cognition* **15**, 709–722 (2006).

47. Fu, Q., Dienes, Z. & Fu, X. Can unconscious knowledge allow control in sequence learning? *Consciousness and Cognition* **19**, 462–474 (2010).

48. Song, S., Howard, J. & Howard, D. Perceptual sequence learning in a serial reaction time task. *Experimental Brain Research* **189**, 145–158 (2008).

49. Destrebecqz, A. *et al.* The neural correlates of implicit and explicit sequence learning: Interacting networks revealed by the process dissociation procedure. *Learning & Memory* **12**, 480–490 (2005).

50. Unoka, Z. *et al.* Intact implicit statistical learning in borderline personality disorder. *Psychiatry Res* **255**, 373–381 (2017).

51. Janacsek, K., Borbély-Ipkovich, E., Nemeth, D. & Gonda, X. How can the depressed mind extract and remember predictive relationships of the environment? Evidence from implicit probabilistic sequence learning. *Progress in Neuro-Psychopharmacology and Biological Psychiatry* **81**, 17–24 (2018).

52. Faul, F., Erdfelder, E., Lang, A. G. & Buchner, A. G*Power 3: A flexible statistical power analysis program for the social, behavioral, and biomedical sciences. *Behavior Research Methods 2007 39:2* **39**, 175–191 (2007).

53. Howard, J. & Howard, D. Age differences in implicit learning of higher-order dependencies in serial patterns. *Psychol Aging* **12**, 634–656 (1997).

54. Hardwick, R. M., Forrence, A. D., Krakauer, J. W. & Haith, A. M. Time-dependent competition between goal-directed and habitual response preparation. *Nature Human Behaviour* **3**, 1252–1262 (2019).

55. Keramati, M., Dezfouli, A. & Piray, P. Speed/accuracy trade-off between the habitual and the goal-directed processes. *PLoS Comput Biol* **7**, e1002055 (2011).

56. Török, B., Janacsek, K., Nagy, D. G., Orbán, G. & Nemeth, D. Measuring and filtering reactive inhibition is essential for assessing serial decision making and learning. *Journal of Experimental Psychology: General* **146**, 529 (2017).
